# Mapping the intellectual landscape of lactylation research: a bibliometric analysis of global trends and emerging frontiers

**DOI:** 10.3389/fphar.2026.1841236

**Published:** 2026-10-08

**Authors:** Jieyan Wang, Zhou Li, Xiya Li, Xiyu Liu, Zhaohua Zhong, Yantong Wan, Hui Liang

**Affiliations:** 1 Department of Urology, The Longhua Affiliated Hospital, School of Medicine, Southern University of Science and Technology, Shenzhen, China; 2 School of Stomatology, Southern Medical University, Guangzhou, China; 3 The First School of Clinical Medicine, Southern Medical University, Guangzhou, China; 4 State Key Laboratory of Cellular Stress Biology, School of Life Science, Faculty of Medicine and Life Sciences, Xiamen University, Xiamen, China

**Keywords:** bibliometric analysis, cancer, epigenetic regulation, immune microenvironment, lactylation, metabolic reprogramming

## Abstract

**Background:**

Lactylation is an emerging post-translational modification (PTM) that links lactate metabolism with epigenetic and protein-level regulation. The rapid expansion of this field has created a need for a systematic overview of its knowledge structure and research frontiers.

**Methods:**

We retrieved 1,231 English articles and reviews from the Web of Science Core Collection and Scopus up to 7 November 2025. Excel, CiteSpace, VOSviewer, and Bibliometrix were used to analyze publication trends, collaboration networks, influential contributors, co-cited references, and keyword evolution.

**Results:**

Annual publications and citations increased sharply after 2019. China and the United States were the two major contributors in publication output and collaboration networks. *Advanced Science* and *Frontiers in Immunology* were prominent publication venues, and authors including Xu K, Zhao YM, Huang H, and Zhang D showed high bibliometric visibility. Co-citation and keyword analyses identified two major research axes: lactylation in cancer biology, tumor immune regulation, and therapy resistance; and lactylation in metabolic reprogramming and disease-associated stress responses.

**Conclusion:**

Lactylation research is moving from site discovery toward mechanistic studies of pathophysiological regulation. The present bibliometric map provides a quantitative framework for understanding the field, while emphasizing that current translational claims remain largely preclinical and require further mechanistic and biomarker validation.

## 1 Introduction

Lactate, the final product of glycolysis, serves not only as a metabolic fuel for various organs but also as a crucial signaling molecule that supports essential physiological processes (Brooks, 2020; Lee, 2021; Li et al., 2022). In 2019, Zhang D et al. reported in *Nature* that lactate can directly promote histone lactylation through a lactate-derived histone lysine lactylation (Kla) mark, thus stimulating gene transcription (Zhang et al., 2019). This revealed a previously unrecognized non-metabolic function of lactate in epigenetic regulation (Zhang et al., 2019; Niu et al., 2024). This discovery formally introduced “lactylation” as a novel post-translational modification (PTM) (Zhang et al., 2019), and subsequent reviews further systematized this concept (Peng and Du, 2025). As a unique PTM, lactylation covalently binds to lysine residues of proteins, which influences gene expression regulation and core cellular physiological processes (Zhang et al., 2019; Niu et al., 2024; Peng and Du, 2025). It plays a central role in tumor metabolic reprogramming linked to the Warburg effect, with lactate dehydrogenase A (LDHA) acting as a key regulatory enzyme in this process (Ji and Xia, 2025; Zhang et al., 2025a; Yang et al., 2025). Currently, research on lactylation has increasingly expanded into the field of disease, as it is closely associated with the pathogenesis of various organ-specific disorders, including cardiac (Zhang et al., 2023; Zhu et al., 2024), neurological (Hagihara et al., 2021; Li et al., 2023), hepatic (Yang et al., 2023; Kotsiliti, 2023), and renal diseases (Wang Y. et al., 2024; Li H. et al., 2025). Additionally, lactylation is relevant across various disease categories, such as oncology (Gu et al., 2022; Li F. et al., 2024), immunological disorders (Huang J. et al., 2024; Fu et al., 2024), and infectious or inflammatory diseases (Yang K. et al., 2022; Liu et al., 2024). Importantly, emerging evidence indicates that lactylation exhibits a “context-dependent dual role” in disease progression, demonstrating both pathogenic and protective effects depending on various pathological conditions (Gong et al., 2024). As research at the interface of metabolism and epigenetics has accelerated, lactylation has emerged as a key modulator of metabolic reprogramming and a focal point in disease-oriented life science research (Chen et al., 2021; Liu et al., 2025; Chen X. et al., 2025).

Bibliometrics, an analytical methodology that combines statistical approaches and quantitative assessment, can systematically map the developmental landscape of disciplines by identifying trends and emerging frontiers (Ninkov et al., 2021). So far, it has been successfully applied across various fields, including innate immunity (Shi et al., 2022; Lai et al., 2023), neurodegenerative disorders (Fu et al., 2023; Meng et al., 2025), and metabolic diseases (Deng et al., 2022; Yang et al., 2024). To date, only a few preliminary bibliometric analyses have been reported on lactylation, and a comprehensive global mapping remains lacking (Li M. et al., 2025). This includes aspects such as publication trends, key contributors, research hotspots, and collaboration networks. This gap hinders researchers from quickly grasping the broader scope of the field and recognizing future potential breakthroughs, while also limiting innovation across teams and disciplines. In response to this issue, this study aims to apply bibliometric methods to the field of lactylation. By systematically analyzing publication trends, core contributors, research hotspots, and collaboration networks, this research seeks to fill the gap in global bibliometric analysis of lactylation. The findings are expected to provide a strategic foundation for future researchers and enhance the precision and efficiency of lactylation research.

## 2 Materials and methods

### 2.1 Data sources

All relevant literature was retrieved from Web of Science Core Collection (WoSCC) and Scopus, two widely used citation databases in bibliometric research with broad coverage of peer-reviewed journals and citation records (Falagas et al., 2008). In WoSCC, TS denotes the Topic Search field. The search strategy employed in this study was as follows: TS = (“lactylation*” OR “lactylated” OR (“lactate modification”) OR (“lysine lactylation”)). In Scopus, the equivalent search strategy was: TITLE-ABS-KEY (“lactylation*” OR “lactylated” OR “lactate modification” OR “lysine lactylation”). The literature search was finalized on 7 November 2025, with article types restricted to “Article” and “Review”, and only English publications were included. Ultimately, a total of 1,231 eligible publications were retrieved, including 966 Research Articles and 265 Reviews, all of which were downloaded in full-text format for subsequent data extraction and analysis. Extracted information included country/region, institution, author, publication journal, citation bursts, and keyword bursts. An overview of the data sources, inclusion criteria, and literature screening process is presented in Figure 1.

**FIGURE 1:**
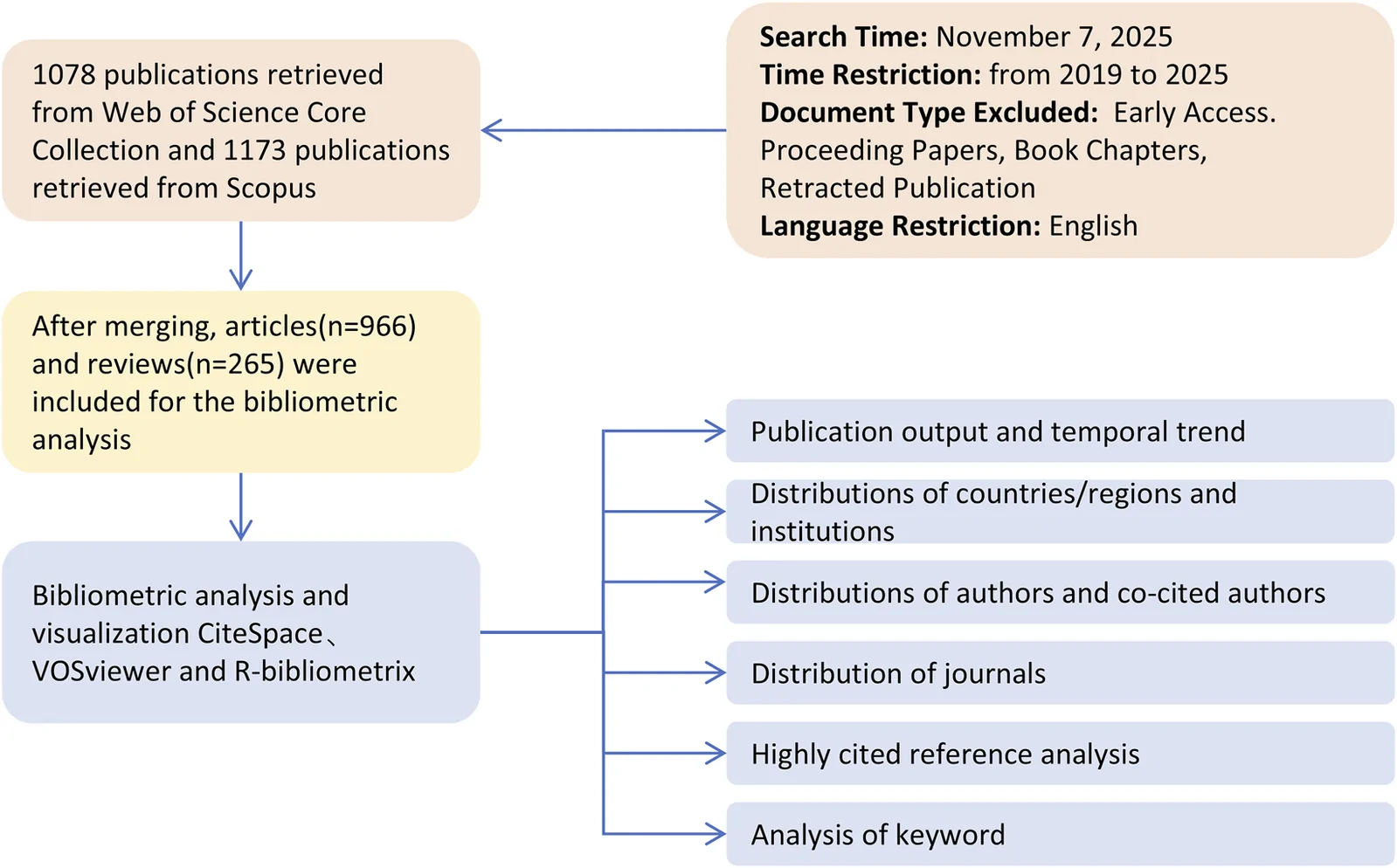
Flowchart of study retrieval and selection.

### 2.2 Data cleaning, analysis and visualization

Prior to formal analysis, we carried out thorough data cleaning and standardization. We standardized author name variations, inconsistent expressions for affiliations, and journal title abbreviations to prevent the occurrence of duplicate or biased statistics. Duplicate records were removed based on unique document identifiers. All cleaning procedures were conducted carefully to ensure data reliability.

The standardized data were imported into CiteSpace (version 6.3 R3), VOSviewer (version 1.6.18), and Bibliometrix for quantitative and visual analysis. CiteSpace was applied with consistent core parameters across analyses: timespan 2019–2025, slice length 1 year, g-index (k = 50), LRF = 3.0, L/N = 10, LBY = 5, e = 1.0, Pathfinder pruning, and log-likelihood ratio (LLR) clustering (Chen, 2006). VOSviewer was used to quantify associations among keywords, authors, journals, countries/regions, and institutions; unless otherwise stated, node size represents frequency or citation count, line thickness represents link strength, and total link strength (TLS) represents the sum of link weights connected to a given node (Van Eck and Waltman, 2010). Furthermore, Bibliometrix, a specialized R package within RStudio, was used to supplement quantitative visualization, with the core data standardization method set to *Z-score* (Aria and Cuccurullo, 2017). Microsoft Excel 2016 was used to depict annual trends in publication output and citation frequencies. Since all datasets were obtained from the publicly available WoSCC and Scopus databases, no ethical approval or institutional review board consent was required.

## 3 Results

### 3.1 Annual trends

To illustrate the developmental landscape of lactylation research, we analyzed annual publication counts and citation frequencies (Figure 2A). This analysis reveals a significant upward trajectory in lactylation research from 2019 to 2025. Following the emergence of the field in 2019, the number of annual publications entered an exponential growth phase, increasing from 308 articles in 2024 to 689 articles by late 2025. This surge in publication output was accompanied by a substantial increase in academic impact, with annual citations peaking at 9,201 in 2024. The cumulative growth of the literature aligns with the logistic growth model, as shown in Figure 2B. The fitting parameters indicate that the field has not yet reached the 50% inflection point of its predicted total capacity, suggesting a phase of sustained expansion. Additionally, Figure 2C identified notable peaks in citation deviation (red line) in 2019 and 2022. These peaks highlight the high citation density of research published during these years, underscoring their importance as critical intellectual foundations for lactylation research.

**FIGURE 2:**
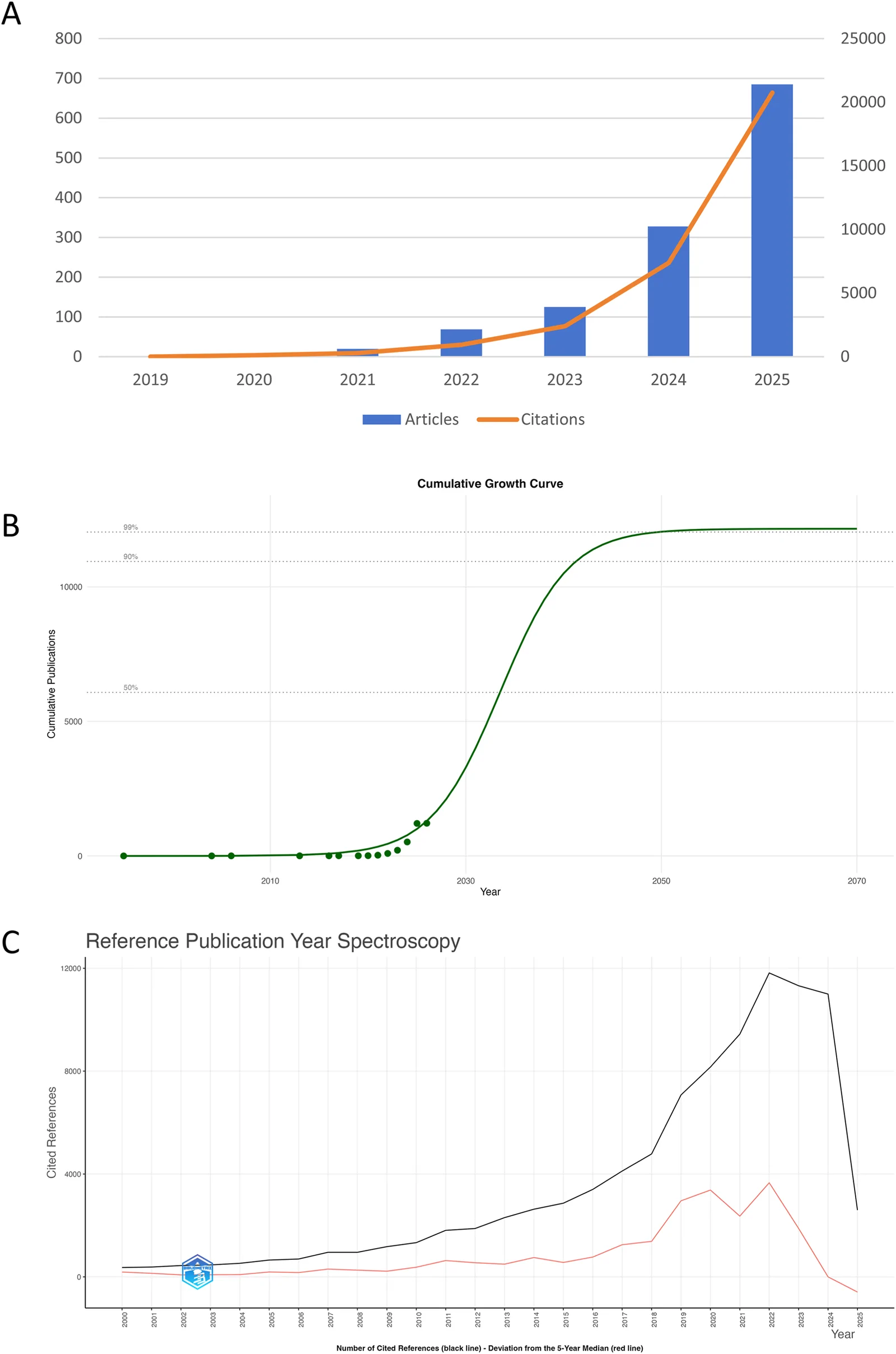
Annual publication trends and growth patterns. **(A)** Annual publication and citation trends in lactylation research from 2019 to 2025. **(B)** Logistic growth curve fitting the cumulative publication volume of lactylation research literature. **(C)** Reference Publication Year Spectroscopy (RPYS) of lactylation research, showing the number of cited references (black line) and their deviation from the 5-year median (red line) by publication year.

### 3.2 Distributions of countries/regions and institutions

We conducted statistical and visual analyses of the retrieved data to examine the geographic distribution, institutional impact, and landscape of international collaboration in the field of lactylation. The results indicate that the global lactylation research landscape is characterized by significant geographic concentration and strong institutional collaboration, with China and the United States acting as the primary driving forces. As shown in Table 1, China ranks first with 1,129 articles, 28,944 citations, and a total link strength (TLS) of 122, significantly surpassing the United States, which is in second place with 94 articles, 7,803 citations, and a TLS of 74. TLS is used as an indicator of collaboration intensity—higher TLS values signify more intense international collaborations and stronger academic linkages. Figure 3A verifies that China has become the global central hub, maintaining dense collaborative networks with North America and Europe. This dominant position is further supported by the Corresponding Author’s Countries analysis (Figure 3B), where China leads with over 1,100 publications. Notably, China has a high proportion of Single Country Publications (SCP) accounting for nearly 86%, indicating a self-sustained domestic research ecosystem. In contrast, the United States has a higher percentage of Multiple Country Publications (MCP), reaching approximately 57%, reflecting its greater focus on international collaborative research. To further explore these structural relationships, the Country Co-occurrence Network (Figure 3C) shows that China and the United States occupy central positions, acting as key bridges that connect distinct collaborative clusters.

**TABLE 1:** The top 10 countries/regions ranked by publications, citations, and total link strength.

Rank	Countries	Documents	Countries	Citations	Countries	TLS
1	China	1129	China	28,944	China	122
2	USA	94	USA	7803	USA	74
3	Germany	13	South Korea	2520	Germany	17
4	Canada	11	Denmark	665	Netherlands	15
5	Japan	11	Germany	617	Canada	14
6	Italy	10	Switzerland	616	Australia	11
7	Netherlands	9	England	429	Switzerland	9
8	Australia	8	Japan	380	Singapore	8
9	England	7	Italy	289	Romania	8
10	South Korea	7	Canada	218	England	7

TLS, total link strength, is defined as the sum of link weights connecting a node with all other nodes in the network.

**FIGURE 3:**
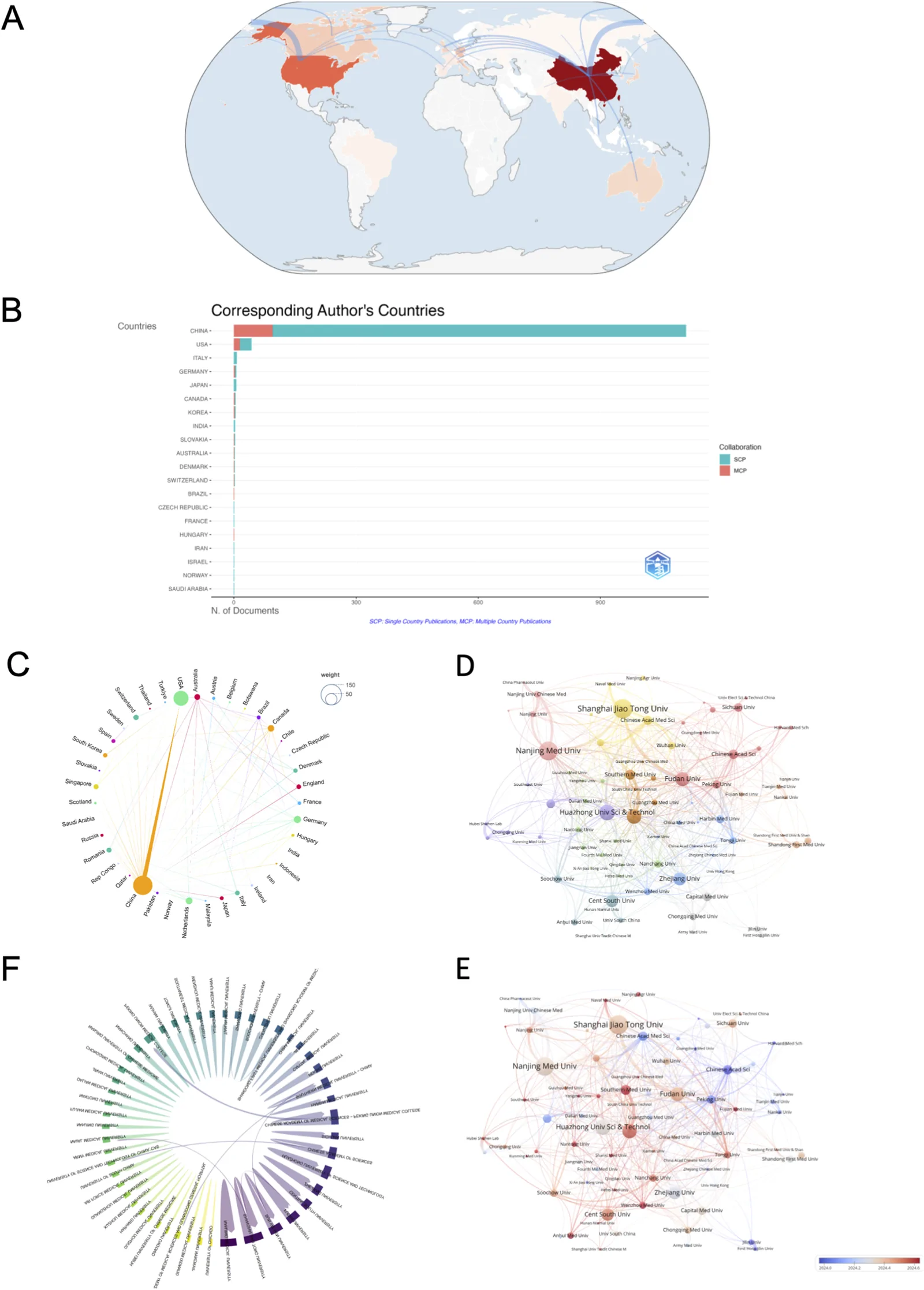
Geographic and institutional distribution of lactylation research output. **(A)** Geographic distribution of lactylation research output across countries/regions. **(B)** Quantitative analysis of publications by corresponding authors’ affiliated countries. **(C)** Country co-occurrence network diagram in lactylation research. **(D)** Collaboration network diagram of the top contributing institutions in lactylation research. **(E)** Institutional collaboration network, with color coding representing the time period of inter-institutional collaboration (January–June 2024). **(F)** Chord diagram of inter-institutional collaboration in lactylation research. The threshold for displayed countries is set to a minimum publication count of 1.

Additionally, we analyzed the contributions of institutions by ranking the top 10 institutions based on publication output and citation counts (Table 2). Our findings show that Shanghai Jiao Tong University leads in publication output with a total of 73 papers, closely followed by Nanjing Medical University with 71 papers. Remarkably, all top 10 institutions in terms of publication output are based in China. In terms of citation frequency, the Chinese Academy of Sciences ranks first with a total of 4,171 citations, followed by the University of Chicago with 3,197 citations. Among the top 10 institutions by citation frequency, the University of Chicago is the only institution from the United States, with all others being Chinese institutions. Collaboration network analysis (Figure 3D) highlights that inter-institutional interactions are mainly concentrated within China, with Shanghai Jiao Tong University and Fudan University serving as key hubs in the collaboration network, while cross-border institutional connections remain relatively limited. Figure 3E reveals that the institutional landscape of lactylation research is continuously evolving. Over the past 6 years, Nanjing Medical University and Shanghai Jiao Tong University have made substantial contributions, whereas Southern Medical University, Wenzhou Medical University, and Anhui Medical University have shown increasing research activity. These patterns indicate expanding institutional participation and collaboration in lactylation research. However, they should be interpreted as bibliometric evidence of growing pre-translational research activity, rather than as evidence of clinically validated lactylation-based biomarkers or therapeutic strategies. The Institutional Collaboration Chord Diagram (Figure 3F) reveals a highly integrated collaborative network, particularly among leading Chinese institutions. This high collaborative intensity is accompanied by an exponential increase in research productivity. The close inter-institutional links and rapid expansion of research capacity underscore the transition of lactylation research from niche mechanistic exploration to a large-scale collaborative research frontier led by major Chinese academic clusters.

**TABLE 2:** The top 10 institutions based on the number of publications and citations.

Rank	Institution	Publications	Original country	Institution	Citations	Original country
1	Shanghai Jiao Tong University	73	China	Chinese academy of sciences	4171	China
2	Nanjing Medical University	71	China	The University of Chicago	3197	United States of America
3	Huazhong University of Science and Technology	57	China	Sichuan University	2888	China
4	Sun Yat-sen University	53	China	Nanjing Medical University	2708	China
5	Fudan University	51	China	Shanghai Jiao Tong University	2523	China
6	Zhejiang University	47	China	Fudan University	1895	China
7	Central South University	45	China	Zhejiang University	1727	China
8	Southern medical University	35	China	Zhengzhou University	1560	China
9	Chinese Academy of Sciences	33	China	Sun Yat-sen University	1532	China
10	Chinese Academy of Medical Sciences	32	China	Peking University	1368	China

To further evaluate publication impact at the country level, we established a journal ranking system based on the 2024 Journal Impact Factor (JIF). Journals were classified into four tiers: Rank A (JIF≥10), Rank B (5≤JIF <10), Rank C (3≤JIF <5), and Rank D (JIF <3). Country-level contribution was assessed using full counting, in which internationally collaborative papers were counted once for each country represented in the author affiliations. We then calculated the JIF-weighted output for each country as the sum of JIF values of the journals in which its papers were published. China showed the highest JIF-weighted publication output, with a total score of 8559.6, followed by the United States (1268.4), Germany (245.7), the United Kingdom (150.9), and Italy (140.3) (Supplementary Figure 1A). These results indicate that China had the strongest overall publication influence in this field when journal impact factor was considered. We further analyzed the distribution of each country’s publications across the JIF-based journal ranks. Although China had the largest total JIF-weighted output, the proportion of publications in Rank A journals differed among countries (Supplementary Figure 1B). Rank A papers accounted for 22.3% of China’s matched publications, compared with 48.4% for the United States, 66.7% for Germany, 50.0% for the United Kingdom, and 40.0% for Italy. These findings suggest that some countries with fewer total publications had a higher relative concentration of papers in high-impact journals. Overall, the JIF-based ranking analysis provides an additional perspective on national publication strength beyond publication counts alone.

### 3.3 Distribution of authors and co-citedauthors

The distribution of author contributions provides valuable insights into the core research capacity and evolutionary trends within the field of lactylation research. A total of 8,326 authors were identified, and their productivity patterns generally conform to Lotka’s Law (Supplementary Figure 2). Notably, the vast majority of authors contributed only a single publication in the field, which is represented by the highest observed data point at the beginning of the log-log scale. This distribution pattern characterizes a rapidly expanding research area with a continuous influx of new researchers. According to the statistical analysis of publication counts and citation frequencies of authors in the field of lactylation research (Supplementary Table 1), the top ten authors with the highest number of publications and total citations were identified. Among them, Xu K from Hubei University of Chinese Medicine ranked first with 11 published papers. In addition, co-citation analysis revealed that Zhang D possesses considerable academic influence in this area, with a total of 1,096 citations and a TLS of 15,993, indicating his central role in shaping the research paradigm within the field. However, publication quantity or citation frequency alone does not fully reflect an author’s academic impact. To provide a more balanced evaluation, we also adopted the *h*-index and *g*-index as complementary metrics in Table 3. The *h*-index measures an author’s productivity and citation impact, representing having h papers each cited at least h times, while the *g*-index gives additional weight to highly cited papers, emphasizing outstanding academic contributions. The results showed that Xu K not only demonstrated prolific research output but also achieved the highest *h*-index (Zhang et al., 2025a) and *g*-index (Zhu et al., 2024), reflecting both sustained research activity and consistent quality in recent years. Meanwhile, Huang H and Zhao YM, although not leading in publication count or total citations, both exhibited high *h*-index values (7 for each) and *g*-index values (8 for Huang and nine for Zhao), signifying their notable and enduring academic influence in the field.

**TABLE 3:** The top 15 authors based on academic impact indicators.

Rank	Author	h_index	g_index	m_index	Citations	Documents	Publication year_start
1	XU KANG	8	11	2	708	11	2022
2	HUANG HE	7	8	1	3030	8	2019
3	ZHAO YINGMING	7	9	1	3104	9	2019
4	LI CHUANFU	6	6	1.5	901	6	2022
5	LI XIN	6	8	2	190	8	2023
6	WANG CHUNLI	6	9	1.5	647	9	2022
7	WANG SHUNSHUN	6	9	2	405	9	2023
8	YANG YI	6	8	2	395	8	2023
9	ZHANG KAI	6	8	1.5	370	8	2022
10	ZHANG YU	6	11	1.5	281	11	2022
11	BAI XUE	5	6	1	382	6	2021
12	CHEN CHEN	5	6	1.25	238	6	2022
13	CHEN WEI	5	6	1.667	431	6	2023
14	LI JIE	5	5	1.667	194	5	2023
15	LI MIN	5	6	1	90	6	2021
16	LI TAO	5	8	2.5	182	8	2024

Author collaborative patterns were visualized using VOSviewer, which identified 13 main collaborative clusters (Figure 4A). Key collaborative hubs include a green cluster centered on Xu K, a red cluster led by Zhao YM, a light blue cluster led by Zhang Y, and a light orange cluster headed by Wang W. The co-citation analysis (Figure 4B) reveals that major clusters centered on Zhang D, Yang ZJ, and Zhang Y exhibit strong interconnections, indicative of an integrated research landscape in the field. To characterize the temporal dynamics of author influence, Figure 4C uses node size and the intensity of red color to denote the extent of each author’s influence. Our results indicate that Zhang Y and Zhao YM exert the strongest comprehensive influence. Meanwhile, the research group led by Ge SL, while positioned peripherally in the network and having a relatively small publication output, demonstrates notable academic impact. As depicted in the temporal distribution of author contributions (Figure 4D), although the field emerged in 2019, it experienced a significant surge in high-quality research output between 2024 and 2025. During this period, core authors witnessed a marked increase in both their publication output and citation frequency. Figure 4E further clarifies the intellectual lineage of lactylation research. This network is centered on Zhang D’s landmark 2019 publication, which serves as the foundational node for the entire research field. Through key transitional nodes including Yu J (2021) and Li WH (2024), this network links early foundational mechanistic discoveries with recent investigations into pathological regulatory functions, thereby establishing a coherent framework for the scientific evolution of lactylation research.

**FIGURE 4:**
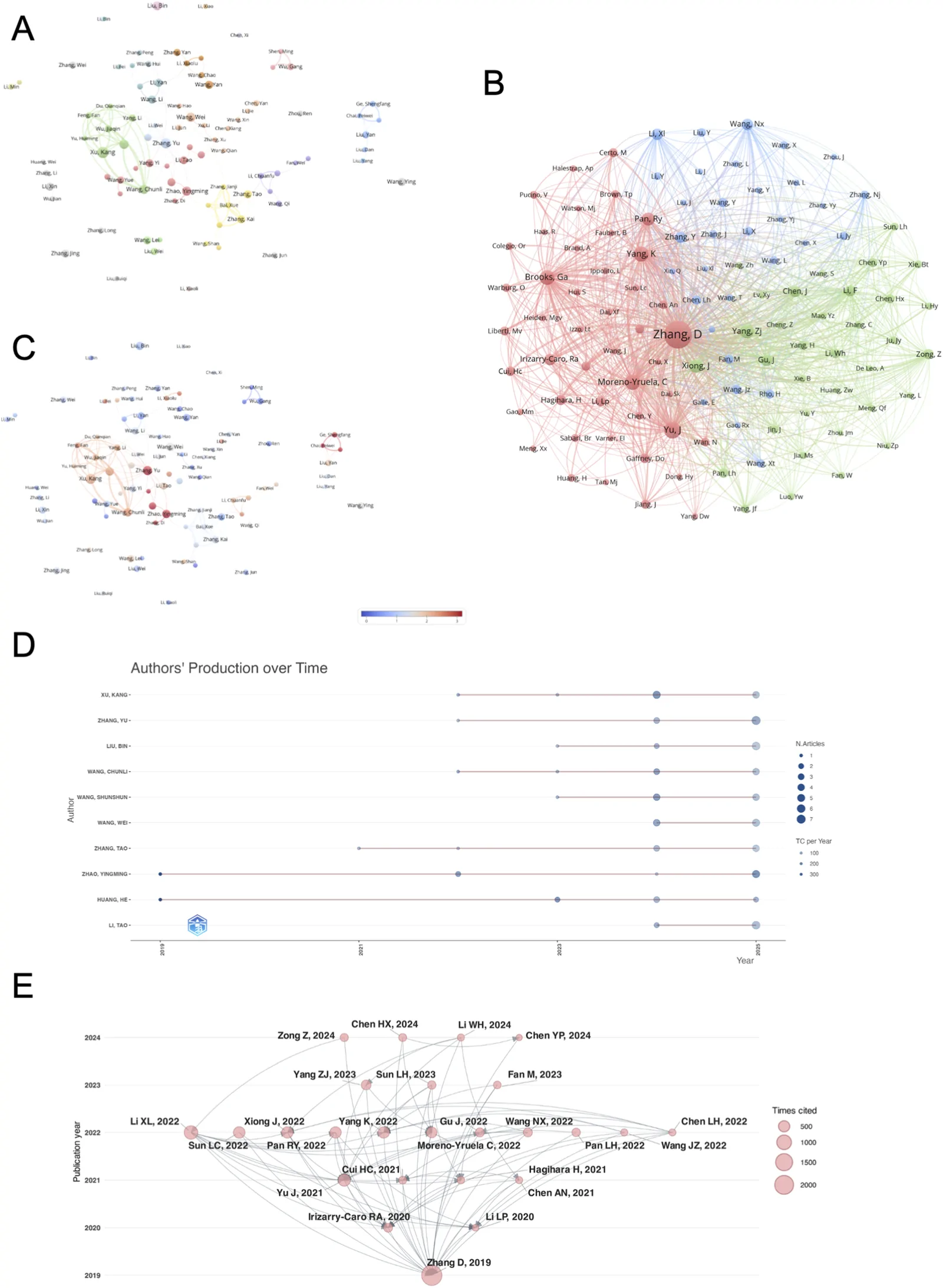
Author collaboration networks and scholarly impact. **(A)** Author collaboration network diagram in lactylation research. **(B)** Co-citation network visualization of core authors in lactylation research. **(C)** Visualization of the influence and centrality of key authors in lactylation research. **(D)** Temporal trends of publication output by the top 10 authors (2019–2025). **(E)** Timeline visualization of the burst detection and academic contribution of key authors in lactylation research. The threshold for displayed authors is set to a minimum publication count of 5. The threshold for displayed co-cited authors is set to a minimum co-citation count of 75.

### 3.4 Distribution of journals and co-cited scholarly periodicals

The landscape of influential journals in lactylation research was evaluated through publication volume and co-citation analysis. As detailed in Table 4, *Advanced Science* led the field with 45 publications, closely followed by *Frontiers in Immunology* (42 papers). While high-output journals reflect current research hotspots, co-citation counts highlight the foundational authority and potential of specific journals (Ioannidis, 2023). Notably, *Nature* recorded the highest co-citation count at 3,178, followed by *Cell Metabolism* (2,201) and *Nature Communications* (Lee, 2021; Zhou et al., 2025). This underscores their crucial roles in shaping the development of lactylation research. All top 10 journals with the highest publication volume and co-citation counts fall within the Q1 quartile of the Journal Citation Reports (JCR). These include prominent titles such as *Nature* (IF = 48.5) and *Cell* (IF = 42.5), reflecting the field’s alignment with top-tier academic publishing standards and the high visibility of lactylation research within the broader scientific community. Further exploration of the structural distribution of these journals through VOSviewer-based clustering analysis identified four distinct clusters (Figure 5A): a blue cluster centered on *Frontiers in Immunology*, a green cluster centered on *Advanced Science*, a red cluster centered on *International Journal of Molecular Sciences*, and a purple cluster centered on *International Immunopharmacology*. Figure 5B uncovers an evolving landscape of journal influence: while the blue cluster centered on *Frontiers in Immunology* dominated in the early stages, the green cluster centered on *Advanced Science* has become increasingly influential in recent years. This shift is further supported by journal co-citation clustering analysis (Figure 5C), which identifies four thematic pillars: immunology, oncology, metabolism and molecular biology, and multidisciplinary sciences. These thematic clusters collectively highlight the critical role of lactylation in metabolic regulation and a wide range of pathophysiological processes. Figure 5D identifies *Advanced Science* and *Frontiers in Immunology* as core literature sources for lactylation research, while a diverse range of peripheral journals continues to drive the overall expansion of the research field. Finally, to characterize the intellectual evolution in the field, we generated a journal dual-map overlay. As shown in Figure 5E, it illustrates a distinct knowledge flow trajectory from cited journals (right) to citing journals (left). Specifically, the intellectual foundation of the field is primarily rooted in Molecular, Biology, and Genetics, with knowledge flows extending to application-focused research domains, including Molecular, Biology, and Immunology and Medicine, Medical, and Clinical sciences. This progression underscores the transition of lactylation research from fundamental biochemical investigations to advanced immune regulatory mechanisms and clinical translational research.

**TABLE 4:** The top 10 journals ranked by publication count and co-citation count.

Rank	Journal	Publications	IF(JCR2024)	JCR quartile	Co-cited journal	Citations	IF(JCR2024)	JCR quartile
1	Advanced science	45	14.1	Q1	Nature	3178	48.5	Q1
2	Frontiers in immunology	42	5.9	Q1	Cell metabolism	2201	30.9	Q1
3	Scientific reports	22	3.9	Q1	Nature communications	2101	15.7	Q1
4	Cancer Letters	21	10.1	Q1	Cell	1979	42.5	Q1
5	International journal of molecular sciences	20	4.9	Q1	Frontiers in immunology	1630	5.9	Q1
6	Journal of translational medicine	20	7.5	Q1	Proceedings of the national academy of sciences of the United States of America	1539	9.1	Q1
7	Nature communications	17	15.7	Q1	Cell reports	1403	6.9	Q1
8	Cell death and disease	16	9.6	Q1	International journal of molecular sciences	1301	4.9	Q1
9	Cell reports	15	6.9	Q1	Molecular cell	1297	16.6	Q1
10	International journal of biological macromolecules	15	8.5	Q1	Science advances	1065	12.5	Q1

**FIGURE 5:**
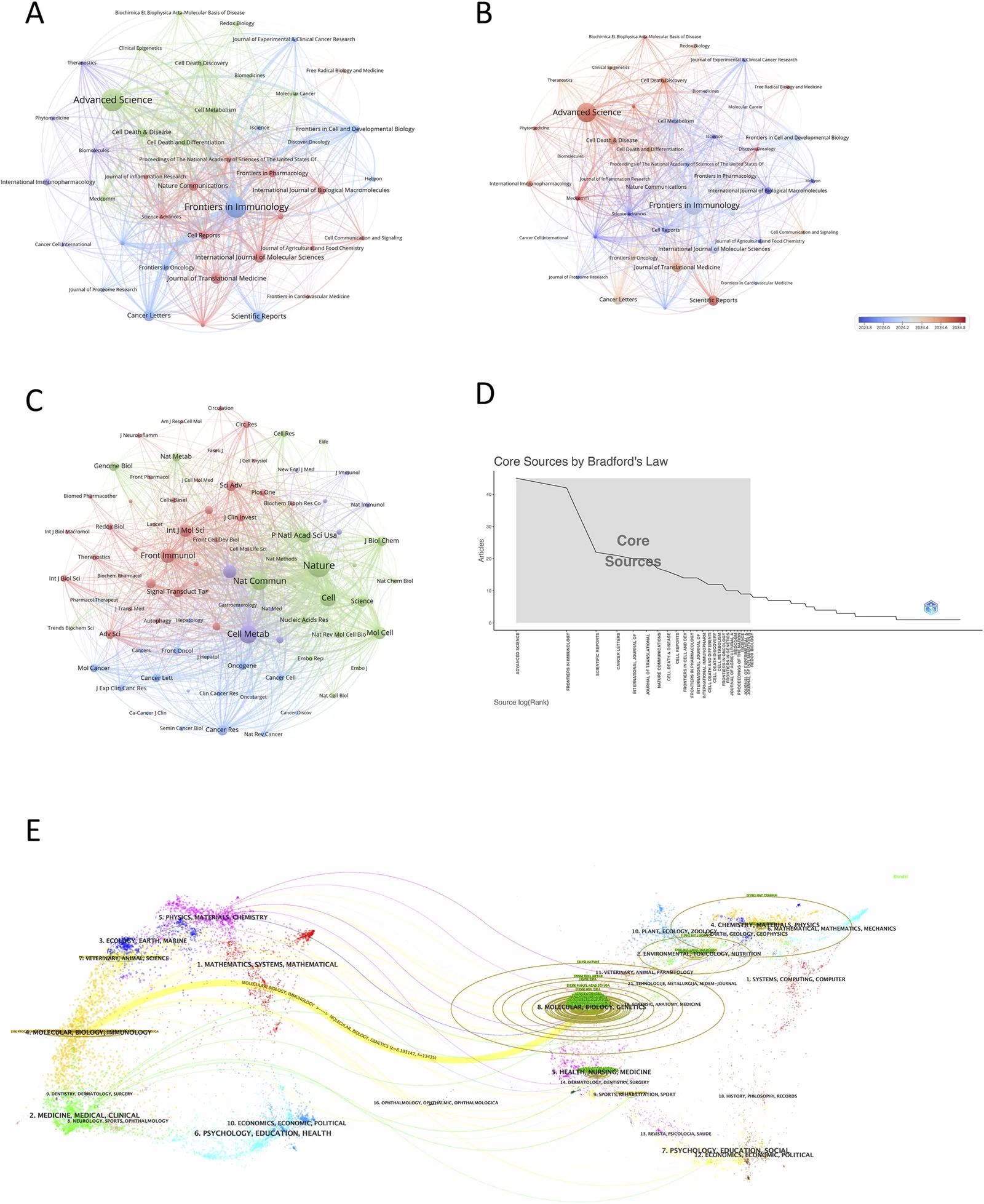
Journal distribution patterns and knowledge flow. **(A)** Co-occurrence network visualization of journals publishing lactylation research. **(B)** Temporal evolution of journal influence, with color indicating publication year (2023.8–2024.8). **(C)** Core journal co-citation network, showing key research clusters. **(D)** Bradford’s Law analysis identifying core journal sources, with the core zone highlighted in grey. **(E)** Dual-map overlay of knowledge flow among subject categories. The threshold for displayed journals is set to a minimum publication count of 7. The threshold for displayed co-cited journals is set to a minimum co-citation count of 200.

### 3.5 Distribution of co-cited references

Analyzing the most highly cited articles helps identify seminal contributions and research trends in the lactylation field. Table 5 lists the 15 most cited publications in the field. The study titled “Metabolic regulation of gene expression by histone lactylation” by Zhang D et al. published in *Nature* (2019) (Zhang et al., 2019), ranks first with 2,484 total citations. This landmark study clarifies the metabolic regulatory role of histone lactylation in gene expression, illustrating how lactate, as a metabolic intermediate, modulates transcriptional activity through histone modification. It lays a crucial foundation for understanding the key role of lactylation in epigenetics and metabolomics. Two subsequent studies of considerable clinical relevance are also among the top cited: one is “Lactate metabolism in human health and disease” by Li et al. (2022) with 848 citations. This comprehensive systematic review discusses the diverse roles of lactate metabolism in human health and disease progression, emphasizing that lactate serves not only as a byproduct of energy metabolism but also as a key regulator in cellular signaling, immune regulation, and disease pathogenesis. It promotes in-depth investigations into lactate-associated physiological and pathological processes. The other is “Histone lactylation drives oncogenesis by facilitating m6A reader protein YTHDF2 expression in ocular melanoma” by Yu et al. (2021) with 627 citations. This research demonstrates that histone lactylation enhances ocular melanoma progression by increasing the expression of the m6A reader protein YTHDF2, shedding light on the epigenetic regulatory role of lactylation in tumorigenesis and offering novel insights for lactylation research in oncology.

**TABLE 5:** The top 15 articles with the most citations.

Rank	Author	Article title	Source title	Cited	Year	Document type	Doi
1	Zhang, D; Tang, ZY; Huang, H; Zhou, GL; Cui, C; Weng, YJ; Liu, WC; Kim, S; Lee, S; Perez-Neut, M; Ding, J; Czyz, D; Hu, R; Ye, Z; He, MM; Zheng, YG; Shuman, HA; Dai, LZ; Ren, B; Roeder, RG; Becker, L; Zhao, YM	Metabolic regulation of gene expression by histone lactylation	NATURE	2484	2019	Article	10.1038/s41586-019-1678-1
2	Li, XL; Yang, YY; Zhang, B; Lin, XT; Fu, XX; An, Y; Zou, YL; Wang, JX; Wang, ZB; Yu, T	Lactate metabolism in human health and disease	SIGNAL TRANSDUCTION AND TARGETED THERAPY	848	2022	Review	10.1038/s41392-022-01151-3
3	Yu, J; Chai, PW; Xie, MY; Ge, SF; Ruan, J; Fan, XQ; Jia, RB	Histone lactylation drives oncogenesis by facilitating m6A reader protein YTHDF2 expression in ocular melanoma	GENOME BIOLOGY	627	2021	Article	10.1186/s13059-021-02308-z
4	Sun, LC; Zhang, HF; Gao, P	Metabolic reprogramming and epigenetic modifications on the path to cancer	PROTEIN and CELL	545	2022	Review	10.1007/s13238-021-00846-7
5	Xiong, J; He, J; Zhu, J; Pan, JL; Liao, WJ; Ye, HY; Wang, HF; Song, YJ; Du, Y; Cui, BJ; Xue, MG; Zheng, WL; Kong, XX; Jiang, K; Ding, KF; Lai, LH; Wang, QQ	Lactylation-driven METTL3-mediated RNA m6A modification promotes immunosuppression of tumor-infiltrating myeloid cells	MOLECULAR CELL	515	2022	Article	10.1016/j.molcel.2022.02.033
6	Pan, RY; He, L; Zhang, J; Liu, XH; Liao, YJ; Gao, J; Liao, Y; Yan, YH; Li, QQ; Zhou, XH; Cheng, JB; Xing, Q; Guan, FX; Zhang, J; Sun, LY; Yuan, ZQ	Positive feedback regulation of microglial glucose metabolism by histone H4 lysine 12 lactylation in Alzheimer’s disease	CELL METABOLISM	511	2022	Article	10.1016/j.cmet.2022.02.013
7	Yang, K; Fan, M; Wang, XH; Xu, JJ; Wang, YN; Tu, F; Gill, PS; Ha, TZ; Liu, L; Williams, DL; Li, CF	Lactate promotes macrophage HMGB1 lactylation, acetylation, and exosomal release in polymicrobial sepsis	CELL DEATH AND DIFFERENTIATION	507	2022	Article	10.1038/s41418-021-00841-9
8	Moreno-Yruela, C; Zhang, D; Wei, W; Bæk, M; Liu, WC; Gao, JJ; Danková, D; Nielsen, AL; Bolding, JE; Yang, L; Jameson, ST; Wong, JM; Olsen, CA; Zhao, YM	Class I histone deacetylases (HDAC1-3) are histone lysine delactylases	SCIENCE ADVANCES	433	2022	Article	10.1126/sciadv.abi6696
9	Yang, ZJ; Yan, C; Ma, JQ; Peng, PP; Ren, XL; Cai, SL; Shen, X; Wu, YC; Zhang, S; Wang, XY; Qiu, SJ; Zhou, J; Fan, J; Huang, H; Gao, Q	Lactylome analysis suggests lactylation-dependent mechanisms of metabolic adaptation in hepatocellular carcinoma	NATURE METABOLISM	369	2023	Article	10.1038/s42255-022-00710-w
10	Gu, J; Zhou, JR; Chen, QY; Xu, XZ; Gao, J; Li, XY; Shao, Q; Zhou, B; Zhou, HM; Wei, S; Wang, Q; Liang, Y; Lu, L	Tumor metabolite lactate promotes tumorigenesis by modulating MOESIN lactylation and enhancing TGF-β signaling in regulatory T cells	CELL REPORTS	314	2022	Article	10.1016/j.celrep.2022.110986
11	Wang, NX; Wang, WW; Wang, XQ; Mang, G; Chen, JF; Yan, XY; Tong, ZH; Yang, QN; Wang, MD; Chen, LQ; Sun, P; Yang, YP; Cui, JX; Yang, M; Zhang, YF; Wang, DN; Wu, J; Zhang, MM; Yu, B	Histone lactylation boosts Reparative gene activation Post-myocardial infarction	CIRCULATION RESEARCH	312	2022	Article	10.1161/CIRCRESAHA.122.320488
12	Irizarry-Caro, RA; McDaniel, MM; Overcast, GR; Jain, VG; Troutman, TD; Pasare, C	TLR signaling adapter BCAP regulates inflammatory to reparatory macrophage transition by promoting histone lactylation	PROCEEDINGS OF THE NATIONAL ACADEMY OF SCIENCES OF THE UNITED STATES OF AMERICA	300	2020	Article	10.1073/pnas.2009778117
13	Sun, LH; Zhang, Y; Yang, BY; Sun, SJ; Zhang, PS; Luo, Z; Feng, TT; Cui, ZL; Zhu, T; Li, YM; Qiu, ZJ; Fan, GJ; Huang, C	Lactylation of METTL16 promotes cuproptosis via m6A-modification on FDX1 mRNA in gastric cancer	NATURE COMMUNICATIONS	276	2023	Article	10.1038/s41467-023-42025-8
14	Pan, LH; Feng, F; Wu, JQ; Fan, SB; Han, JJ; Wang, SX; Yang, L; Liu, WQ; Wang, CL; Xu, K	Demethylzeylasteral targets lactate by inhibiting histone lactylation to suppress the tumorigenicity of liver cancer stem cells	PHARMACOLOGICAL RESEARCH	275	2022	Article	10.1016/j.phrs.2022.106270
15	Chen, HX; Li, Y; Li, HF; Chen, XC; Fu, HF; Mao, DL; Chen, W; Lan, LX; Wang, CM; Hu, KS; Li, J; Zhu, CM; Evans, I; Cheung, E; Lu, DN; He, YL; Behrens, A; Yin, D; Zhang, CH	NBS1 lactylation is required for efficient DNA repair and chemotherapy resistance	NATURE	262	2024	Article	10.1038/s41586-024-07620-9

Furthermore, we constructed a document co-citation network using CiteSpace to characterize the foundational knowledge in the lactylation field. As shown in Figure 6A, nineteen major clusters were identified in the network, with Cluster #0 (proteomics) occupying the most central position. While early research concentrated on fundamental metabolic topics, such as Cluster #10 (lactate), the network indicates a clear research expansion toward clinical pathologies, including Cluster #1 (cardiovascular disease), Cluster #8 (prostate cancer), and Cluster #6 (temozolomide resistance). Figure 6B illustrates the relational mapping among cited references, contributing authors, and core keywords. The analysis confirms that Zhang D’s 2019 foundational study remains the most influential co-citation source, serving as the primary knowledge node for the entire field. The flow lines indicate that this seminal reference, along with key works like that of Yu J (2021), is primarily supported by a core group of active researchers, including Wang W, Zhang Y, and Liu B. These researchers effectively connect fundamental epigenetic concepts, such as “histone lactylation”, with downstream biological processes like “glycolysis” and “metabolic regulation”, bridging the gap between basic and translational research. Additionally, temporal shifts in research focus were quantified through citation burst analysis (Figure 6C). Among the top 25 references, Zhang D (2019) exhibits the highest burst strength (163.34), with its influential period extending from 2020 to the present. Notably, studies by Xiong J (2022) and Dai XF (2022) exhibit sustained citation bursts extending into 2025, underscoring their crucial role in shaping the conceptual framework that links lactate metabolism, epigenetic regulation, and tumor biology. This pattern suggests that the field is evolving beyond the initial discovery of lactylation toward more mechanistic and translational themes.

**FIGURE 6:**
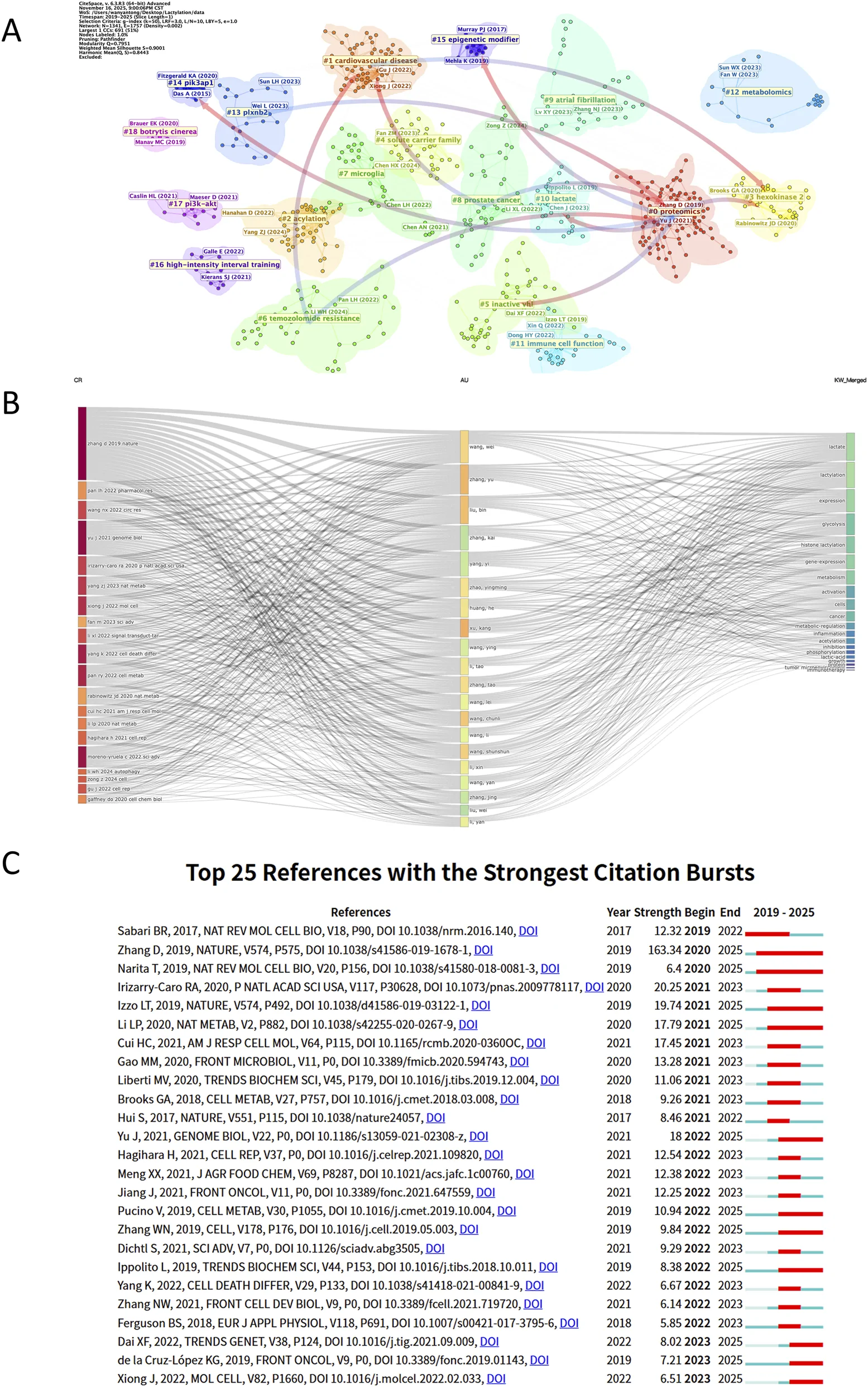
Reference co-citation networks and citation bursts. **(A)** Reference co-citation network clusters, with node size indicating citation frequency. **(B)** Triple coupling network visualization of cited references, core authors, and keywords. **(C)** Top 25 references with the strongest citation bursts (2019–2025).

### 3.6 Distribution of keywords

As concise summaries of research content, keywords provide insights into the research frontiers and developmental trends of lactylation research. Table 6 presents the top 20 high-frequency keywords in this field, where “lactylation” ranks first with 689 occurrences and a TLS of 1,206, followed by “lactate” (208 occurrences, TLS = 458) and “glycolysis” (113 occurrences, TLS = 241). Notably, terms related to oncology including “tumor microenvironment”, “cancer”, and “hepatocellular carcinoma” feature prominently, underscoring the close association between lactylation research and tumor biology. Using VOSviewer, we visualize the correlation and frequency of these keywords in Figure 7A. “Lactylation” occupies the central node position, surrounded by closely associated core terms such as “glycolysis”, “lactate”, “post-translational modifications”, “tumor microenvironment”, and “immunotherapy”. Subsequent temporal evolution analysis (Figure 7B) shows that the early network was dominated by dense core clusters centered around “post-translational modification” and “lactate”. In contrast, the more recent phase displays a shift toward keywords such as “ferroptosis”, “machine learning”, “acute kidney injury”, “immune infiltration”, and “drug resistance”. While tumor-related research historically dominated the field, the currently influential keywords shown in Figure 7C cluster primarily around emerging mechanistic intersections such as “ferroptosis” and “mitophagy”, as well as non-oncological breakthroughs in “acute kidney injury” and therapeutic resistance mechanisms such as “pd-l1”.

**TABLE 6:** Top 20 keywords based on occurrences and total link strength.

Rank	Keyword	Occurrences	TLS	Rank	Keyword	Occurrences	TLS
1	lactylation	689	1206	11	Macrophage	36	74
2	Lactate	208	458	12	Ferroptosis	31	49
3	Glycolysis	113	241	13	Metabolism	30	73
4	Post-translational modification	109	244	14	Machine learning	28	65
5	Epigenetic	51	128	15	Epigenetic modification	26	61
6	tumor microenvironment	49	136	16	Inflammation	26	67
7	Metabolic reprogramming	47	125	17	Prognosis	25	51
8	Cancer	45	111	18	Histone	24	50
9	Immunotherapy	45	111	19	Lactate metabolism	21	54
10	Hepatocellular carcinoma	44	87	20	Sepsis	20	46

TLS, total link strength, is defined as the sum of link weights connecting a node with all other nodes in the network.

**FIGURE 7:**
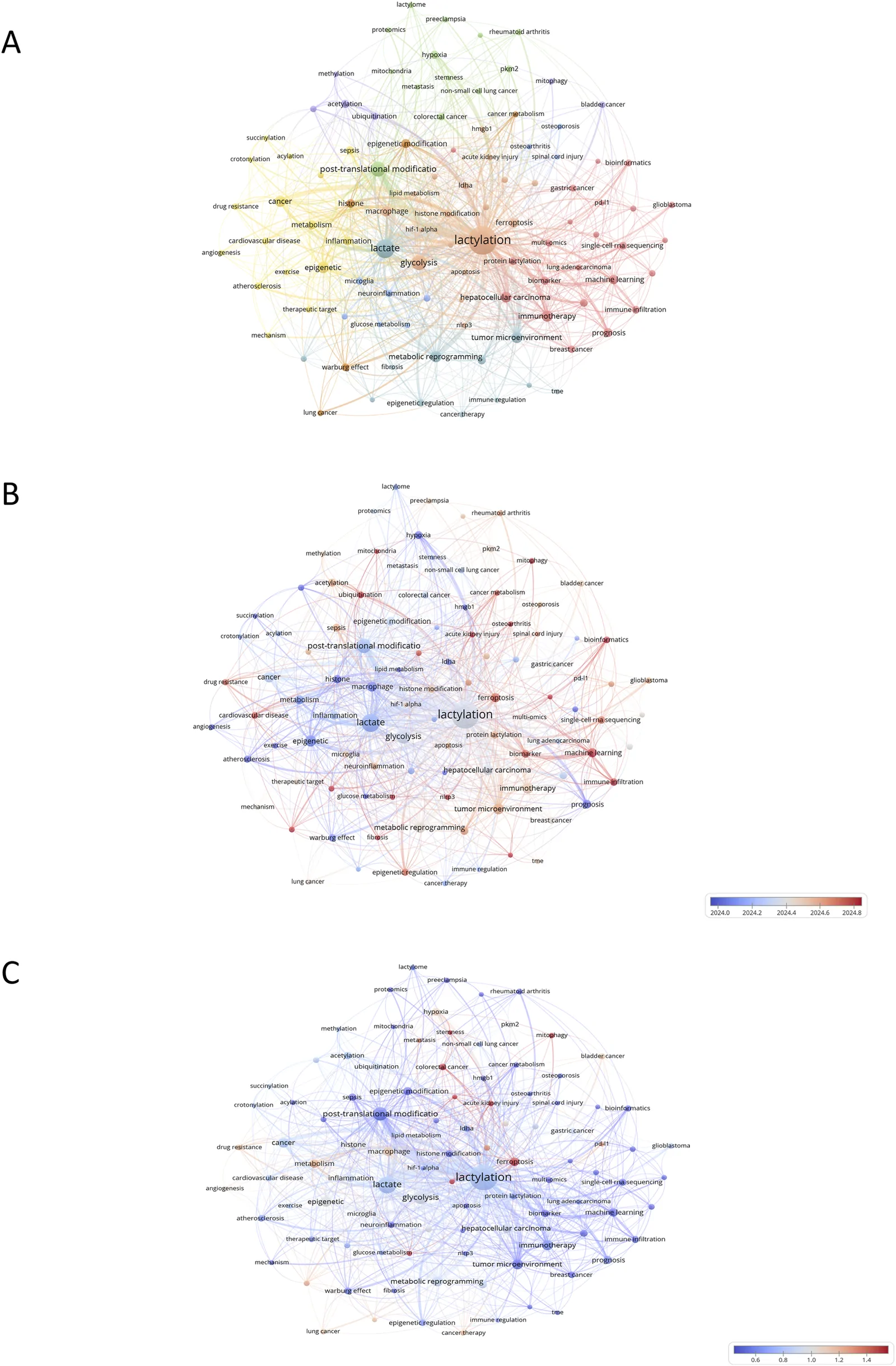
Keyword co-occurrence patterns and temporal evolution. **(A)** Co-occurrence network visualization of high-frequency keywords in lactylation research. **(B)** Temporal evolution of keyword co-occurrence networks (January–August 2024), with color indicating publication year. **(C)** Visualization of keyword association strength in lactylation research, color-coded by co-occurrence intensity (0.6–1.4). The threshold for displayed author keywords is set to a minimum occurrence count of 6.

For knowledge domain structural analysis, Figure 8A illustrates a distinct spatial distribution comprising three primary clusters. The first focuses on fundamental biochemical mechanisms, represented by terms including “lactylation”, “glycolysis”, and “ferroptosis”. The second centers on clinical and translational applications, particularly “immune infiltration” and the development of “prognostic models”. The third connects lactylation with classical PTMs, such as “acetylation” and “ubiquitination”. This thematic classification is further validated by the hierarchical clustering analysis in Supplementary Figure 3, which generates a statistical dendrogram reflecting these term associations. The thematic prominence and developmental maturity of these research topics in 2025 are depicted in the thematic map (Figure 8B). Themes including “tumor microenvironment”, “immunotherapy”, and “hepatocellular carcinoma” are located in the upper-right quadrant, categorizing them as “Motor Themes” with high centrality and density that drive the current development of the field. In contrast, “post-translational modification,” “acetylation,” and “ubiquitination” are classified as “Niche Themes”, while “metabolic reprogramming” and “epigenetic” remain “Basic Themes” serving as the essential foundational framework for the entire discipline. From a temporal evolutionary perspective, Figure 8C traces the dynamic development of lactylation research from 2019 to 2025, revealing a transition from “histone lactylation” and “macrophage” function toward broader molecular mechanisms such as “metabolic reprogramming” and “protein lactylation”, as well as new disease domains like “acute kidney injury”. This evolutionary shift is further supported by trend topic analysis in Supplementary Figure 4, which demonstrates a migration of research interest from initial studies on “fungal pathogenicity” and basic biochemical topics toward more systemic regulatory mechanisms. Regarding thematic intensity and frontier identification, Figure 8D reveals that “lactylation” has exhibited a strong increase since 2021, a trend that has continued through 2025. Notably, the recent emergence of keywords such as “machine learning” reflects the growing integration of computational biology approaches into lactylation-related data mining, while the top 25 keyword burst analysis in Supplementary Figure 5 provides detailed parameters for the rise and decline of various subfields. Finally, correlation analysis of average publication year and normalized citation rate reveals current research dynamics (Figure 8E). Keywords in the upper right corner, including “central nervous system”, “immune evasion”, “colorectal cancer”, and “acute kidney injury”, suggest increasing pre-translational interest in disease mechanisms, biomarker exploration, and therapeutic hypothesis generation.

**FIGURE 8:**
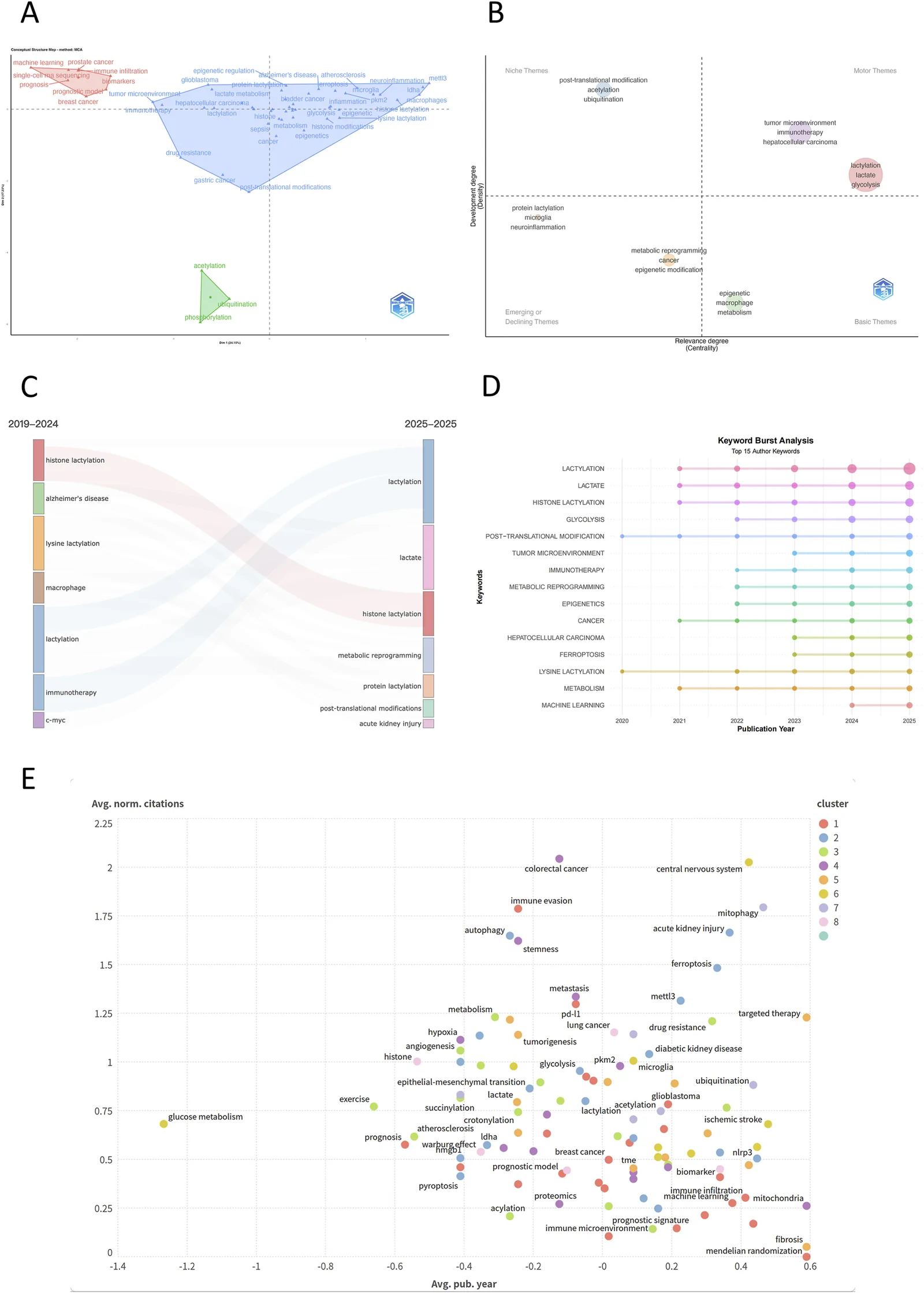
Keyword cluster analysis and research theme dynamics. **(A)** Cluster analysis of core research keywords in lactylation research. **(B)** Thematic map of research topics in lactylation field based on centrality and density. **(C)** Sankey diagram of research theme migration in lactylation research (2019–2024–2025). **(D)** Burst detection analysis of core keywords in lactylation research. **(E)** Scatter plot visualization of the relationship between average publication year and average normalized citation rate for lactylation research keywords, with clusters indicated by color.

## 4 Discussion

### 4.1 Global research trends of lactylation

To characterize the developmental trajectory of lactylation research, we performed a comprehensive bibliometric analysis of publications since the field’s emergence. In 2019, Zhang D et al. published a landmark study in *Nature* (Zhang et al., 2019) that identified lactate-derived histone Kla as a novel epigenetic modification and established a direct regulatory axis linking cellular metabolism with gene expression and epigenetics. Prior to this, lactate had been mainly investigated as a glycolytic metabolite and a metabolic or signaling regulator (Brooks, 2020; Lee, 2021; Li et al., 2022), whereas its role in directly modulating chromatin structure and transcription through a specific PTM remained unrecognized (Xu et al., 2025; Jian et al., 2025). This groundbreaking research shifted the conventional perspective on lactate biology (Zhang et al., 2019) and, together with subsequent reviews, formally positioned lactylation as an independent research topic at the intersection of metabolism and epigenetic regulation (Liu et al., 2025; Lu et al., 2024). Such a conceptual shift has sparked a sustained surge in academic interest (Li M. et al., 2025). Our bibliometric results, particularly those derived from the logistic growth model (Figure 2B), confirm that lactylation research is currently in a phase of rapid growth and has not yet reached the 50% inflection point. This trend implies that while the early mechanistic explorations spurred by citation peaks in 2019 and 2022 are gradually maturing, the field remains far from saturated; instead, it is broadening to encompass a wider biological landscape where lactylation is treated as a universal regulatory signal that mediates cellular adaptation (Liu et al., 2025; Lu et al., 2024). Overall, these observations support the notion that lactylation constitutes a dynamic and highly promising frontier in biomedical research, with significant potential for further development (Liu et al., 2025; Xu et al., 2025; Lu et al., 2024).

Nevertheless, this global expansion of research exhibits significant geographic differences. China has become the leading contributor in terms of publication output, encompassing all top 10 highly productive institutions and maintaining a relatively independent research ecosystem evidenced by high SCP rates. This pattern suggests that lactylation research in China is being driven by a domestically anchored network of laboratories that benefit from sustained investment in epigenetics and omics-oriented funding landscape (Li D. et al., 2025; National Natural Science Foundation of China, 2018). Additionally, there is a rapid integration of lactylation into research programs focused on tumor metabolism and immune-related studies at major academic medical centers (Ji and Xia, 2025; Li et al., 2026). In contrast, although the United States has published fewer articles, it showcases a high ratio of MCP and notable citation impacts from institutions like the University of Chicago. This indicates a collaboration-intensive model in which lactylation is frequently incorporated into multi-institutional and international research programs. Interestingly, changes over time in institutional influence (Figure 3E) indicate a trend toward greater academic diversification. Early pioneers such as Nanjing Medical University and Shanghai Jiao Tong University are now being joined by emerging research centers like Southern Medical University. Many of these rising institutions have developed strong research programs focusing on studying lactate-driven metabolic reprogramming, immune regulation, and organ injury, which helps explain their rapid increase in prominence within the collaboration network (Ji and Xia, 2025; Li et al., 2026; Cheng et al., 2025). This shift in institutional leadership aligns with the thematic evolution observed in our keyword analysis, where the focus on lactylation has gradually expanded from oncology-focused work to broader areas such as inflammation, autoimmunity, and chronic metabolic diseases (Xu et al., 2025). However, international academic interactions still appear to be largely restricted to regional clusters. The persistence of these regionally constrained structures implies that current collaboration patterns may limit the exchange of mechanistic and translational insights among research communities. Future advancements in this field will likely depend on breaking through these regionally limited networks and enhancing global cooperation to better understand the complex roles of lactylation in biological systems (Evran et al., 2025).

The intellectual lineage of this field, visualized through author collaboration and co-citation networks (Figure 4), reveals a well-defined landscape of academic influence. The field is anchored by the foundational work of Zhang D and the team led by Zhao YM, whose 2019 study in *Nature* (Zhang et al., 2019) remains the most co-cited and is considered a central node in the research framework. This seminal work provided direct epigenetic evidence for the functional role of lactate in gene regulation (Zhang et al., 2019), and subsequent studies have significantly expanded the framework of metabolic-epigenetic crosstalk (Merkuri et al., 2024; Zhang et al., 2025b). By identifying histone Kla, the study established a specific biochemical link between glycolytic flux and chromatin state, offering a more nuanced understanding of the biological significance of the Warburg effect beyond simple energy production (Zhang et al., 2019; Zhang et al., 2025a). The subsequent growth of the field has been driven by a core group of academics focused on both mechanistic depth and disease relevance. Among them, Xu Kang stands out as one of the most prolific contributors in our dataset (Table 3), representing the clinical forefront of the field. His research group has substantially broadened the preclinical dimensions of lactylation by elucidating its roles across diverse pathological models (Singh et al., 2025). In oncology, they have shown that histone H3 lactylation promotes stemness remodeling in hepatocellular carcinoma stem cells (Feng et al., 2024) and that natural products such as royal jelly acid and fargesin can inhibit tumor progression by targeting specific lactylation sites (H3K9la and H3K14la) or metabolic enzymes like PKM2 (Xu et al., 2023). Beyond cancer, their studies in cardiovascular disease indicate that Lumican-induced H3 lactylation, along with p300-dependent regulation, drives calcific aortic valve disease (Huang Y. et al., 2024; Wang C. et al., 2024). Additionally, work on intervertebral disc degeneration has revealed that glutamine can protect against disc degeneration by inhibiting glycolysis and reducing AMPKα lactylation, thus promoting autophagy and limiting cellular senescence (Zhang Y. et al., 2024). In parallel with these clinically targeted advances, the biochemical infrastructure and evolutionary scope of lactylation have been further enriched by diverse mechanistic studies. For example, HBO1 has been identified as a lysine lactyltransferase that catalyzes histone lactylation, particularly at H3K9la (Niu et al., 2024), thereby providing a mechanistic basis for site-specific transcriptional control and linking lactylation directly to gene regulation. Further studies have characterized CobB-associated lactylation in *Escherichia coli* (Dong et al., 2022) and demonstrated that Numb/Parkin-directed mitochondrial fitness in cancer cells can drive lactate production, histone lactylation, and tumor cell plasticity (He et al., 2023), thereby extending the relevance of lactylation from prokaryotic metabolic regulation to eukaryotic mitochondrial adaptation. Collectively, these investigations illustrate how Chinese institutions have played a leading role in advancing lactylation research from conceptual discovery to translationally relevant disease models. The structural characteristics of the research community also provide insights into the field’s developmental stage (Wedell et al., 2022). Notably, our analysis reveals that most authors have published only a single paper in this domain. This high proportion of single-contribution authors is a hallmark of a rapidly growing research hotspot, reflecting a significant influx of cross-disciplinary investigators from metabolism, immunology, and oncology who are exploring lactylation within their respective areas of expertise (Cheng et al., 2024). While this influx fosters rapid innovation and thematic diversification (Fagan et al., 2018; Feng and Kirkley, 2020), it also indicates that a stable, highly clustered core community is still in the formation process (Wedell et al., 2022; Cugmas et al., 2016). The simultaneous increase in both publication intensity and academic influence during 2024–2025 indicates that lactylation research has progressed beyond niche theoretical observation. It is now recognized as a significant PTM with the potential to influence cell fate and systemic pathophysiological outcomes, comparable in mechanistic complexity to classical modifications such as phosphorylation and acetylation (Jian et al., 2025; Xu et al., 2024).

A comprehensive analysis of journal distribution and knowledge trajectories further supports the progressive evolution of lactylation research. There is a significant disparity between high-output journals and high-impact co-cited periodicals, reflecting their distinct roles in the development of this field. High-publication journals such as *Advanced Science* and *Frontiers in Immunology* serve as primary hubs for disseminating current research hotspots, especially those focusing on the intersection of metabolic reprogramming and immune-related translational applications. In contrast, the high co-citation counts for journals like *Nature* and *Cell* underscore that the intellectual foundation of the field is still anchored by foundational breakthroughs. This distinction suggests that while specialized high-output journals drive the rapid expansion of research frontiers, the academic legitimacy and core paradigm shifts associated with lactylation are endorsed by these prestigious multidisciplinary periodicals. Furthermore, the observation that all top 10 journals in terms of publication volume and co-citation frequency are categorized in JCR Q1 provides strong evidence for the high academic standing of the field. This concentration reflects the successful transition of lactylation from a niche biochemical discovery to a major research paradigm, attracting high-quality contributions and maintaining high visibility within the broader scientific community (Ji and Xia, 2025; Xu et al., 2025). The dual-map overlay (Figure 5D) and the evolving influence of specific journal clusters (Figure 5B) provide a visual narrative of the “bench-to-bedside” trajectory in this field. The distinct knowledge flow originating from “Molecular, Biology, and Genetics” and extending toward “Immunology, Medicine, and Clinical” applications indicates that lactylation is increasingly viewed as a complex regulatory interface that dictates cell fate and remodels the immune microenvironment (Ji and Xia, 2025; Chen et al., 2021; Zhu Z. et al., 2025). The transition of influence from early-phase immunological focus such as *Frontiers in Immunology* to more recent multidisciplinary and translational focuses like *Advanced Science* further highlights the maturation of this research field. This interdisciplinary integration not only reinforces the broad relevance of lactylation across diverse pathophysiological conditions but also establishes a solid theoretical and experimental basis for its translation into precision medicine and targeted therapeutic strategies (Lu et al., 2024; Zha et al., 2024; Chen J. et al., 2025).

### 4.2 Analysis of lactylation research hotspots

#### 4.2.1 Hotspots of lactylation

The landscape of lactylation research, as illustrated by our co-citation and keyword analysis, demonstrates a remarkable evolution from basic biochemical discovery to integrated investigations of complex pathological processes (Liu et al., 2025). A notable 2019 study by Zhang D et al. in *Nature* (Zhang et al., 2019) identified histone Kla derived from lactate as a novel epigenetic mark. This finding establishes a critical link between cellular metabolism and gene regulation, demonstrating that lactate can directly influence transcriptional activity through histone lactylation (Zhang et al., 2019). Our network visualization (Figure 6) confirms that this landmark discovery remains central to the current framework of the field. At present, lactylation research exhibits a distinct dual-pathway development pattern. The first pathway focuses on cancer pathogenesis, emphasizing how lactylation orchestrates the interaction between tumor cells and the immune environment at a mechanistic level (Ji and Xia, 2025; Li Q. et al., 2025). The second pathway revolves around metabolic reprogramming, exploring lactylation as a widespread metabolic regulator that influences the progression of various diseases, including inflammatory disorders and tissue injury models (Liu et al., 2025; Sun et al., 2025). This research framework is particularly well-established in oncology, where “tumor microenvironment” and “immunotherapy” are identified as major motor themes. This trend reflects a movement from simply identifying lactylation sites to dissecting the complexities of immune microenvironment remodeling (Ji and Xia, 2025; Li Q. et al., 2025). Moreover, the recent emergence of keywords such as “acute kidney injury,” “ferroptosis,” and “mitophagy” indicates that the field is gradually expanding beyond the tumor-centric focus characteristic of its early development. This diversification into non-neoplastic diseases implies that lactylation-associated metabolic remodeling is becoming an important paradigm for interpreting multi-organ pathological mechanisms (Liu et al., 2025; Xu et al., 2025). Meanwhile, emerging studies on histone and non-histone protein lactylation, notable work by Dai XF (2022) (Dai et al., 2022) and Xiong J (2022) (Xiong et al., 2022), have defined one of the current cutting-edge directions in the field, aiming to clarify how lactylation alters epigenetic and post-transcriptional regulation to shape therapeutic resistance and immune evasion within the tumor microenvironment. Finally, the growing prevalence of terms such as “machine learning” and “prognostic models” indicates the increasing integration of computational biology with large-scale metabolic and transcriptomic datasets (Li et al., 2025a). This pattern, however, should be interpreted as an emerging analytical tendency rather than evidence that lactylation has entered clinical practice. Most current prognostic models are generated from public transcriptomic datasets and lactylation-related gene sets, and few have been prospectively validated or linked to direct measurement of site-specific protein Kla. Therefore, our bibliometric results indicate a transition from biochemical site discovery to disease-oriented, mechanism-driven, and preclinical modeling (Chen X. et al., 2025; Fan et al., 2025). At the current stage, the most realistic contribution of lactylation research is to clarify how lactate-derived PTMs shape pathophysiological states and therapy-relevant cellular phenotypes, rather than to provide immediately actionable precision-medicine tools (Gong et al., 2026; Li H. et al., 2024).

#### 4.2.2 Mechanistic integration of lactylation in cancer biology

In our keyword analysis, high-frequency core terms such as “tumor microenvironment”, “cancer”, “immunotherapy”, “metabolic reprogramming”, and “hepatocellular carcinoma” were strongly represented (Table 6). Notably, “tumor microenvironment” and “hepatocellular carcinoma” emerged as motor themes in the strategic diagram (Figure 8B), indicating that cancer-related lactylation has become a mature and influential area within the current disciplinary landscape (Chen J. et al., 2025). These bibliometric patterns should not be read as merely quantitative dominance. Mechanistically, they indicate that cancer is the biological setting in which lactate production, chromatin remodeling, immune suppression, and treatment resistance currently converge most clearly. Tumor cells exposed to hypoxia and oncogene-driven glycolysis generate lactate-rich niches; lactate then functions not only as a carbon metabolite but also as a substrate and signal for histone and non-histone Kla. Recent work summarized by Gong et al. places this glycolysis-lactate-lactylation axis within cancer metabolism and immunotherapy, supporting our bibliometric observation that tumor microenvironment, immunotherapy, and metabolic reprogramming form a highly connected knowledge cluster (Gong et al., 2026). Nevertheless, this relationship is context-dependent: lactylation may function as a driver, an amplifier, or an adaptive consequence depending on tumor lineage, lactate flux, enzymatic context, and the substrate or lysine site involved (Li H. et al., 2024).

At the immune interface, lactylation promotes tumor immune evasion through both tumor-cell-intrinsic and immune-cell-mediated routes. In tumor cells, H3K18la has been shown to activate the POM121/MYC/PD-L1 axis in non-small cell lung cancer, thereby enhancing immune escape (Zhang C. et al., 2024), and LDHB-mediated histone lactylation can activate PD-L1 in ovarian cancer (Hu et al., 2025). In head and neck squamous cell carcinoma, malignant-cell H3K9la induces IL-11 expression and contributes to CD8^+^ T-cell dysfunction and poor immunotherapy response through JAK2-STAT3 signaling (Wang R. et al., 2024). On the immune-cell side, lactate-associated epigenetic remodeling can impair CD8^+^ T-cell and NK-cell tumoricidal functions (Hao et al., 2024; Brand et al., 2016), reprogram pro-inflammatory Th17 cells toward a Treg-like phenotype (Lopez et al., 2022), and promote METTL3-dependent immunosuppressive programs in tumor-infiltrating myeloid cells (Xiong et al., 2022). These examples explain why “immune infiltration”, “PD-L1”, and “immunotherapy” emerge together in the keyword network: lactylation is increasingly studied as a metabolic-epigenetic mechanism that tunes immune checkpoints, effector-cell exhaustion, and suppressive myeloid/Treg states.

Lactylation also contributes to metabolic plasticity rather than acting only as a terminal readout of glycolysis. A self-reinforcing loop can be established in which oncogenic glycolysis increases lactate accumulation, lactate increases Kla, and Kla-dependent transcriptional or protein remodeling further stabilizes glycolytic and stress-adaptive programs. In pancreatic ductal adenocarcinoma, positive feedback between glycolysis and histone lactylation promotes oncogenesis (Li F. et al., 2024). In breast cancer, KCNK1 activates LDHA and increases H3K18la, linking lactate production to changes in cell stiffness, adhesion, proliferation, and metastasis (Hou et al., 2024). Non-histone lactylation broadens this model by modifying metabolic enzymes, transcription factors, and DNA repair proteins, thereby coupling extracellular nutrient stress and intracellular lactate flux to phenotypic adaptation. This provides a mechanistic basis for the co-occurrence of “glycolysis”, “metabolic reprogramming”, “tumor microenvironment”, and “drug resistance” in our maps.

Therapy resistance represents a third major mechanistic axis. Recent studies show that lactylation can protect cancer cells from therapy-induced DNA damage by strengthening homologous recombination repair. MRE11 lactylation at K673 promotes DNA end resection and homologous recombination, whereas inhibition of CBP- or LDH-dependent MRE11 lactylation sensitizes tumor models to chemotherapy and PARP inhibition (Chen Y. et al., 2024). NBS1 lactylation similarly supports efficient DNA repair and contributes to chemotherapy resistance (Chen H. et al., 2024). Beyond DNA repair, long-term enzalutamide exposure increases SLC4A4 expression in prostate cancer; the NF-kappaB/STAT3/SLC4A4 axis mediates p53 lactylation and promotes enzalutamide resistance and prostate cancer progression (Chen H-J. et al., 2024). These findings strengthen the interpretation that lactylation links metabolic plasticity with therapeutic resistance. However, they also reveal that resistance mechanisms are substrate-, tumor-, and treatment-specific, so a general “pan-lactylation” therapeutic strategy is unlikely to be adequate without site-, cell type-, and therapy-specific validation.

Accordingly, the translational implications of lactylation should be framed cautiously. The current evidence supports lactylation as a set of candidate biomarkers and mechanistic vulnerabilities, but not yet as a clinically established therapeutic class. Several barriers remain. First, specificity is unresolved because elevated lactate or pan-Kla signals may reflect hypoxia, inflammation, glycolytic flux, tissue injury, or stromal remodeling rather than a tumor-specific lesion. Second, druggability remains challenging because the known writer and eraser machinery overlaps with broader acylation pathways, including p300/CBP, HBO1, HDAC1-3, and sirtuins, raising concerns about target selectivity and pleiotropic effects (Niu et al., 2024; Moreno-Yruela et al., 2022). Third, measurement remains a limitation because antibody-based pan-Kla assays and gene-set surrogates cannot fully resolve site-specific stoichiometry, substrate identity, or functional causality. Fourth, context-dependency must be considered because the same lactate-lactylation axis can support repair, immune suppression, differentiation, inflammatory resolution, or tissue remodeling depending on the biological setting (Gong et al., 2024; Li H. et al., 2024) (Figure 9).

**FIGURE 9:**
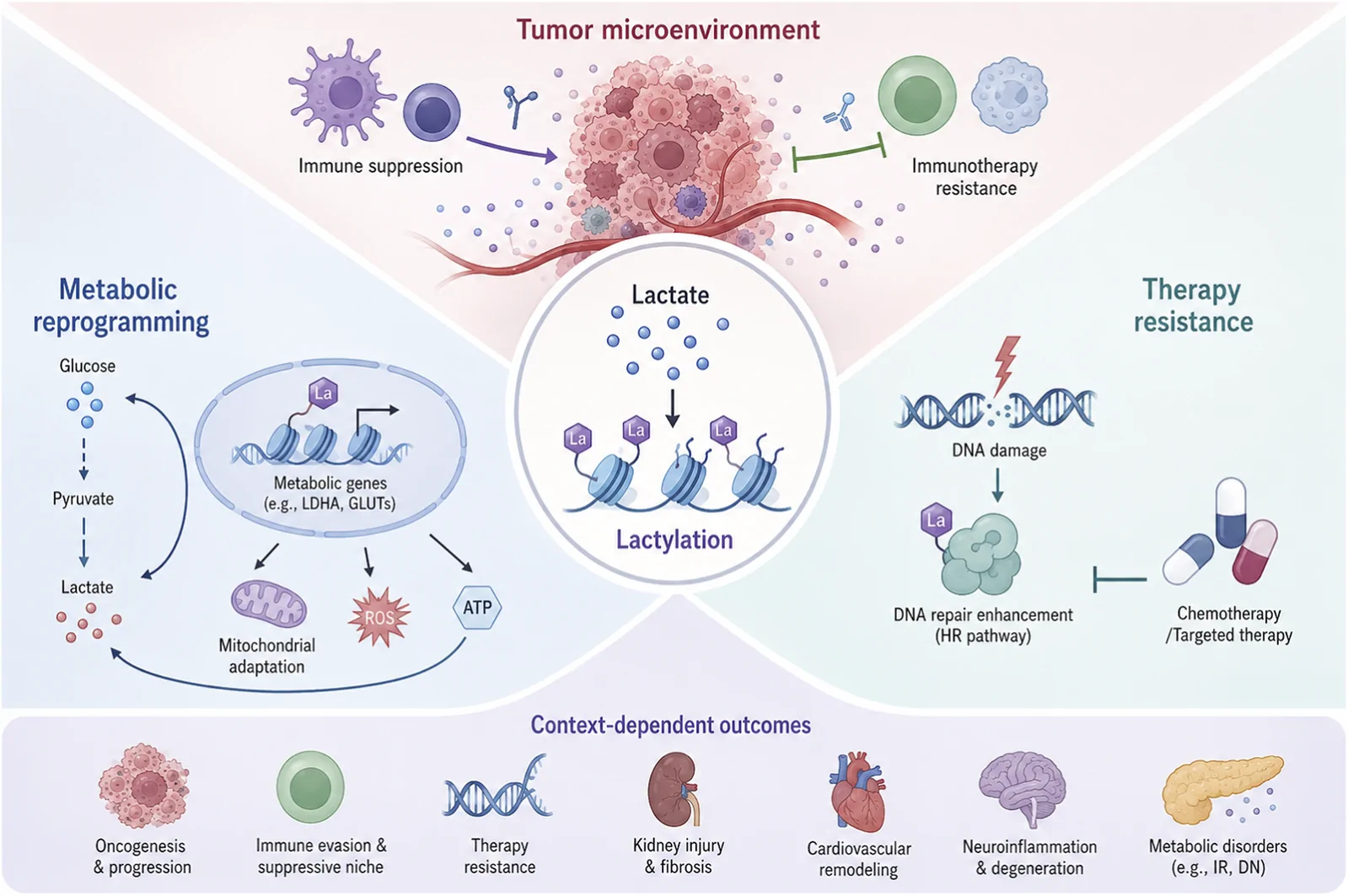
Mechanistic integration of lactylation in cancer biology and metabolic reprogramming.

#### 4.2.3 Lactylation and metabolic reprogramming

Our analyses of keyword evolution (Figure 7B) and impact (Figure 7C) show that emerging metabolism-related terms such as “ferroptosis” and “mitophagy” have gained significant academic influence and prominence. This trend suggests that lactylation research is increasingly focusing on cutting-edge topics involving metabolic reprogramming-mediated regulation of cell fate and stress adaptation (Wang et al., 2023; Lin et al., 2025). In addition, thematic evolution analysis (Figure 8C) indicates a gradual shift in research focus from the initial identification of specific protein modifications to more integrated metabolic regulatory networks, which is consistent with previous studies that position lactylation as an important bridge linking metabolic alterations to disease phenotypes (Wang et al., 2023).

Recent findings demonstrate that lactylation plays a vital role in metabolic reprogramming (Wang et al., 2023; Liu et al., 2022). Building on the aforementioned lactate-rich tumor microenvironment, elevated lactate concentrations serve not only as metabolic byproducts but also as signaling molecules that mediate epigenetic regulation (Brooks, 2020; Lee, 2021; Li et al., 2022; Zhang et al., 2019). Specifically, lactate facilitates covalent histone modifications that alter chromatin architecture, which in turn modulates the transcription of metabolic genes, particularly those encoding glycolytic enzymes such as LDHA and components related to glutamine metabolism (Wang et al., 2023; Zhang J. et al., 2025). In this way, lactylation translates dynamic metabolic flux into relatively stable transcriptional programs that reinforce altered metabolic states. Essentially, the glycolytic surge driven by tumor metabolic reprogramming results in lactate accumulation, which raises histone lactylation levels. These lactylation-dependent chromatin changes, in turn, enhance metabolic reprogramming, establishing a canonical self-reinforcing “metabolism–epigenetics–metabolism” cycle (Yang J. et al., 2022). For instance, histone lactylation promotes the transcription of metabolic genes, including key glycolytic enzymes like LDHA, thereby further amplifying lactate production (Li F. et al., 2024). This self-sustaining “metabolism-epigenetics-metabolism” circuit contributes to tumor progression by driving invasion, metastasis, and immunosuppressive niche maintenance (Ye et al., 2026). Similar positive feedback loops have been documented in multiple cancer models, underscoring that glycolysis-lactate-lactylation axes constitute a core paradigm of malignant metabolic reprogramming (Chen J. et al., 2025; Wang et al., 2023). Elevated extracellular lactate is also internalized by stromal cells, where histone lactylation triggers chromatin remodeling to epigenetically activate profibrotic signaling pathways such as TGF-β/Smad (Fan et al., 2025; Wang et al., 2023), thereby promoting the differentiation of fibroblasts into cancer-associated fibroblasts (CAFs) (Kalluri, 2016; Bhagat et al., 2019). Factors secreted by CAFs like IL-6 boost cancer cell glycolysis, further increasing lactate production and creating a positive feedback loop in which cancer cells and stromal cells mutually reinforce each other’s malignant phenotypes (Wang et al., 2023). Similarly, in clear cell renal cell carcinoma, histone lactylation (H3K18la) mediates a comparable positive feedback loop (Yang J. et al., 2022). Taken together, these observations illustrate that lactylation orchestrates reciprocal metabolic crosstalk between cancer cells and stromal components, thereby stabilizing a protumorigenic microenvironment (Lv et al., 2025; Tan et al., 2025). In addition to its role in metabolic coordination, lactylation-driven regulatory processes also play a significant role in immune modulation (Lv et al., 2025). Notably, lactate-induced epigenetic remodeling is not confined to histone marks but also involves RNA m^6^A and other post-transcriptional layers, as exemplified by the METTL3-dependent immunosuppressive pathways (Xiong et al., 2022; Ye et al., 2026). This integrated view reinforces the concept that lactylation operates at the interface of metabolism and gene regulation to coordinate multi-layered cellular responses, rather than acting in isolation on any single pathway (Wang et al., 2023).

Beyond cancer pathology, lactylation-mediated metabolic reprogramming is increasingly recognized as a fundamental driver in a wide range of non-neoplastic inflammatory and degenerative conditions. In acute kidney injury models, lactate accumulation caused by glycolytic surges in damaged tubular epithelial cells leads to histone lactylation, which epigenetically activates pro-inflammatory and pro-fibrotic signaling (Li et al., 2025b) and accelerates the transition from acute injury to chronic kidney disease. In cardiovascular disease, recent studies indicate that lactylation may participate in post-infarction metabolic and immune remodeling, potentially affecting myocardial repair and pathological remodeling by regulating macrophage phenotypes and fibroblast activation (Zhu et al., 2024; Ma et al., 2017). This regulatory axis extends to neurodegenerative conditions; in Alzheimer’s disease, microglial lactate accumulation drives H3K18la-NF-κB activation, leading to the upregulation of senescence-associated secretory phenotype factors, including IL-6 and IL-8, that accelerate cerebral aging and amyloid pathology (Wei et al., 2023). Furthermore, systemic metabolic disorders exhibit similar lactylation-driven pathophysiology (Zhou et al., 2025): Kla induced by lactate overload worsens insulin resistance (Ma et al., 2022), contributes to the progression of diabetic nephropathy (Chen J. et al., 2024), and promotes vascular calcification (Zhu Y. et al., 2025). Mechanistically, these examples support a conserved model in which metabolic reprogramming engages lactylation to establish self-reinforcing circuits that drive disease progression through coordinated epigenetic regulation, phenotypic plasticity, and microenvironmental remodeling across multiple pathologies (Zhu Z. et al., 2025; Li et al., 2025b; Zhou et al., 2025). Such cross-pathological mechanisms suggest that lactylation-associated metabolic hubs may provide a hypothesis-generating framework for future therapeutic exploration, rather than a universal therapeutic strategy at the current stage (Zhu Z. et al., 2025; Li et al., 2025b; Zhou et al., 2025).

Unlike the oncology-restricted focus outlined above, the findings summarized here highlight a broader, cross-disease perspective in which lactylation operates as a pan-regulatory epigenetic process. Keyword evolution (Figure 7B) and thematic mapping (Figure 8B) consistently indicate that lactylation research has transitioned toward a multi-organ, cross-pathology framework encompassing renal injury, cardiovascular remodeling, and neurodegenerative disorders. This shift conceptually extends lactylation from a tumor-centric modifier to a candidate metabolic-epigenetic mechanism shared across multiple disease contexts. However, this cross-disease relevance remains primarily pre-translational and requires tissue-, cell type-, substrate-, and site-specific validation. (Chen Z. et al., 2025) (Figure 9).

## 5 Limitations

This study provides valuable insights into the development and research trends of lactylation; however, several limitations should be acknowledged. First, although both the WoSCC and Scopus were used, relevant studies indexed only in other databases such as PubMed, Embase, or regional databases may still have been missed, which could introduce selection bias and affect the completeness of the dataset. Future analyses could incorporate additional databases to improve coverage and representativeness. Second, restricting the search to English-language publications may have caused language bias by excluding potentially relevant studies published in other languages. Including multilingual sources in future work may provide a more comprehensive global perspective. Third, bibliometric analyses based on citation counts and co-occurrence patterns have inherent methodological limitations and may underrepresent emerging topics, newly published studies, or interdisciplinary research that has not yet accumulated citations. Applying advanced text-mining or machine-learning techniques may help address this issue. Finally, because the dataset was collected on 7 November 2025, publications released later in the year were not included, which may have influenced the assessment of the most recent research trends. Continuous data updates in future studies would help maintain temporal accuracy.

Another limitation arises from the search-field design. Our retrieval strategy relied on WoSCC topic fields and Scopus TITLE-ABS-KEY fields, which increases specificity for lactylation-focused literature but may miss studies in which lactylation was detected as an ancillary PTM in proteomic or lactylomic datasets but was not mentioned in the title, abstract, or keywords. Consequently, the dataset may underrepresent publications in which lactylation is biologically relevant but not the central focus of the article. Future bibliometric studies could combine title/abstract/keyword retrieval with full-text mining, curated proteomic database searches, and manual screening of supplementary lactylome datasets to reduce this omission bias.

## 6 Conclusion

This study systematically synthesizes current research trends and hotspots in lactylation research. Since 2019, publication output and citation frequency have increased rapidly. China and the United States represent the two major contributors in the global collaboration network, while journals such as Advanced Science and Frontiers in Immunology have served as major publication venues. Considering publication output, citation frequency, h-index, and co-citation structure, Xu K, Zhao YM, Huang H, and Zhang D show high bibliometric visibility, with Zhang D’s 2019 report remaining a foundational co-citation node. Co-occurrence and trend analyses identified two major research directions: one focused on cancer biology, tumor immune regulation, and therapy resistance, and the other focused on metabolic reprogramming and disease-associated stress responses. Overall, lactylation research remains at a pre-translational stage. Although bibliometric trends indicate increasing disease-oriented and therapeutic interest, clinical application will require site-specific functional validation, standardized detection methods, prospective biomarker studies, and rigorous therapeutic evaluation.

## Data Availability

The original contributions presented in the study are included in the article/Supplementary Material, further inquiries can be directed to the corresponding authors.
