# Supplementary material for: Mapping the intellectual landscape of lactylation research: a bibliometric analysis of global trends and emerging frontiers
Source: Front Pharmacol. 2026 Oct 8;17:1841236. doi: 10.3389/fphar.2026.1841236 (PMC13687382; doi:10.3389/fphar.2026.1841236)
Supplement: Supplementary file 1 [file Supplementaryfile1.docx]

Supplementary Material

# 1 Supplementary Figures and Tables

## 1.1 Supplementary Figures

**Figure S1. Country-level publication influence based on 2024 Journal Impact Factor.**

**(A)** JIF-weighted publication output of the top contributing countries. Country-level output was calculated using full counting, in which each internationally collaborative paper was assigned once to every country represented in the author affiliations. The JIF-weighted output was defined as the sum of 2024 Journal Impact Factor values across all matched papers for each country. **(B)** Distribution of matched papers across JIF-based journal ranks for countries with at least five matched papers. Journals were classified into four tiers according to their 2024 JIF: Rank A, JIF ≥ 10; Rank B, 5 ≤ JIF < 10; Rank C, 3 ≤ JIF < 5; and Rank D, JIF < 3. Stacked bars indicate the percentage composition of each country’s matched papers across the four journal ranks.

**Supplementary Figure 2.** Author productivity distribution fitted to Lot’s Law. Author productivity distribution for 8,326 researchers in lactylation research, plotted on a log–log scale. Blue dots indicate the observed proportion of authors with a given number of publications, and the red dashed line shows the theoretical distribution predicted by Lotka’s Law. A sharp peak at x = 1 indicates that most contributors published only one paper in this field, reflecting a large transient researcher population.

**Supplementary Figure 3.** Hierarchical clustering dendrogram of high-frequency keywords. Hierarchical clustering dendrogram of high‑frequency keywords in lactylation research, generated using the Ward.D method. The horizontal dashed line denotes the clustering threshold used to define major research themes. Identified clusters cover core topics including glycolysis, histone lactylation, post‑translational modification, cancer progression, and macrophage polarization, revealing the structural organization of the field.

**Supplementary Figure 4.** Evolution of research topics in lactylation. Temporal evolution of key research topics in lactylation. Circle position on the x‑axis indicates the median publication year, circle size reflects term frequency, and horizontal bars represent the duration of topic prevalence. Early research focused on specific molecular targets and mechanisms, whereas recent studies highlight lactate‑driven epigenetic and metabolic regulation. The persistent prominence of lactylation and glycolysis underscores their fundamental roles in the field.

**Supplementary Figure 5.** Top 25 keywords with the strongest citation bursts (2019–2025). Keywords with the strongest citation bursts over the study period. Red horizontal bars mark the interval during which each keyword exhibited a surge in citations. Histone modification and histone acetylation displayed the earliest and strongest bursts, representing the foundational stage of lactylation research. Recent bursts in lactate‑related terms and immune‑associated keywords identify emerging frontiers and active research hotspots.

## 1.2 Supplementary Tables

**Supplementary Table 1** The top 10 authors based on publications, co-citations, and total link strength

| Rank | Author | Documents | Citations | Countries/regions | institution | Author | co-citations | Total link strength | Countries/regions | institution |
| --- | --- | --- | --- | --- | --- | --- | --- | --- | --- | --- |
| 1 | Xu, Kang | 11 | 708 | China | Hubei University of Chinese Medicine | Zhang, Di | 1096 | 15993 | China | The University of Chicago |
| 2 | Zhang, Yu | 10 | 236 | China | The Second Hospital of Jilin University | Yu, Jie | 458 | 8701 | China | Shanghai Jiao Tong University |
| 3 | Zhao, Yingming | 9 | 3104 | USA | The University of Chicago | Moreno-Yruela, Carlos | 380 | 8954 | Denmark | University of Copenhagen |
| 4 | Wang, Chunli | 9 | 647 | China | Hubei University of Chinese Medicine | Yang, Kun | 372 | 8301 | China | East Tennessee State University |
| 5 | Wang, Shunshun | 9 | 405 | China | Hubei University of Chinese Medicine | Brooks, Ga | 366 | 9240 | USA | University of California, Berkeley |
| 6 | Wang, Wei | 9 | 210 | China | Fudan University | Yang, Zijian | 327 | 7390 | China | Fudan University |
| 7 | Liu, Bin | 9 | 206 | China | The First Hospital of Jilin University | Pan, Rui-Yuan | 295 | 6084 | China | Beijing Institute of Basic Medical Sciences |
| 8 | Huang, He | 8 | 3030 | China | Zhejiang University | Xiong, Jia | 289 | 6750 | China | Zhejiang University |
| 9 | Zhang, Jing | 8 | 594 | China | Beijing Normal University | Irizarry-Caro, Ricardo A. | 240 | 4906 | USA | University of Texas Southwestern Medical Center at Dallas |
| 10 | Yang, Yi | 8 | 395 | China | East China University of Science and Technology, Optogenetics and Synthetic Biology Interdisciplinary Research Center | Gu, Jian | 235 | 6988 | China | Nanjing Medical University |
